# Theory of Nonadiabatic Tunneling Splitting

**DOI:** 10.1021/acs.jpclett.5c00443

**Published:** 2025-05-08

**Authors:** Leonardo Raso, Michele Ceotto, Eli Pollak

**Affiliations:** † Dipartimento di Chimica, Universitá degli Studi di Milano, via C. Golgi 19, 20133 Milano, Italy; ‡ Chemical and Biological Physics Department, 34976 Weizmann Institute of Science, 76100 Rehovoth, Israel

## Abstract

Estimating tunneling splittings is a long-standing quantum mechanical challenge for theoretical methods. Sometimes splittings are so small, i.e., within a fraction of a wavenumber, pushing the limits of experimental detection and computational precision. Currently, most computational methods are able, at best, to obtain only ground-state tunneling splittings, either for symmetric or asymmetric potentials. In this Letter, we introduce a unified theoretical approach, based on a two-state approximation that can be equally applied to symmetric and asymmetric diabatic potential crossing and for excited states, providing reliable estimates even for states near the energy crossing. The method opens the door to analytic approximations for the tunneling splitting of model potential systems. It provides a framework for the introduction of vibrational perturbation theory to the estimation of nonadiabatic tunneling splittings. It also provides new insight into the semiclassical theory, leading to an instanton based steepest descent expression applicable also to excited states. Numerical tests on model systems are promising, providing the groundwork for implementation to future multidimensional applications.

## Graphical abstract

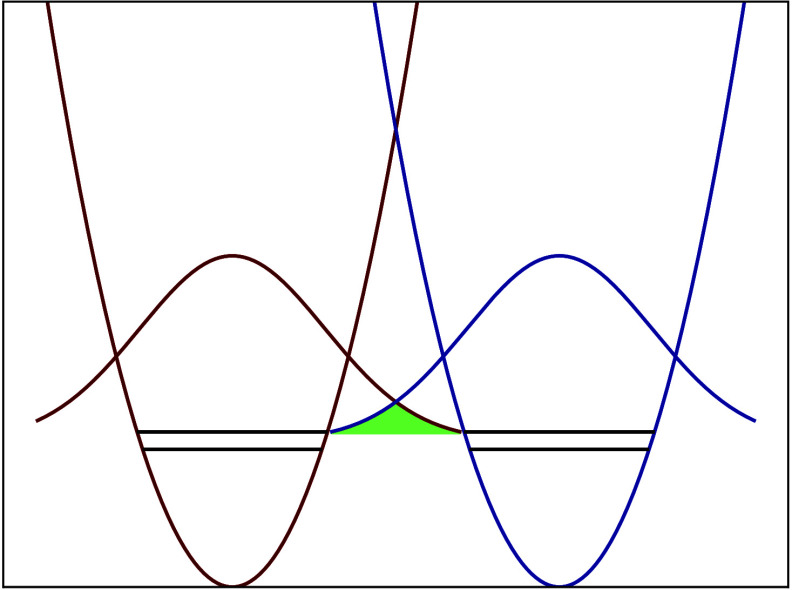

Understanding nonadiabatic-tunneling-induced energy splitting in symmetric and asymmetric systems^1–3^ presents challenges on the fundamental and practical level to this very day. In contrast to adiabatic reaction rates,^4^ there are very few, if at all, analytic results available for nonadiabatic tunneling splitting energies. Many algorithms have been developed for the approximate computation of nonadiabatic-induced reaction rates,^5,6^ but much less so, when considering the nonadiabatic induced tunneling splitting energy. Unraveling the effect of asymmetry on the energy level structure has also been challenging.^7–13^ The semiclassical theory has been developed to some extent,^7,8^ but questions remain. The Euclidean tunneling action in the symmetric tunneling splitting case is only over half a period, while in the asymmetric case it is over the whole period.^7^ How does one go smoothly from symmetric nonadiabatic tunneling to asymmetric?^14^ The ground-state tunneling splitting in the symmetric case may be obtained in practice from thermodynamic considerations, taken to the limit of zero temperature.^12,15^ The Herring formula^16^ has been used to obtain tunneling splitting of excited-state doublets especially for adiabatic tunneling on a single adiabatic double well potential.^9,13,17^ Semiclassical methods for excited states have been developed by Benderskii and Vetoshkin,^18^ but not much has been done when considering nonadiabatic-induced tunneling splitting, especially in the context of excited states. Vibrational perturbation theory has led to a practical method for computing doublet splitting in the adiabatic case,^19,20^ but it has not been applied to doublet splitting induced by nonadiabatic coupling. Can this be remedied?

In this Letter, we use a rather simple but rather accurate theoretical framework to answer many of these questions. Limiting the discussion to two coupled diabatic states, we show that a two-state approximation leads to relatively accurate analytic solutions, to a perhaps novel understanding of the semiclassical theory of tunneling and to extension of vibrational perturbation theory to nonadiabatic induced tunneling splitting of both ground and excited states. The two-state approximation is not limited to weak nonadiabatic coupling and does not make use of the Fermi golden rule in its derivation.

We consider a system with two orthogonal electronic states, such that the Hamiltonian has the form

1 $$Ĥ={Ĥ}_{1}\left|L\rangle \langle L\right|+{Ĥ}_{2}\left|R\rangle \langle R\right|+\mathrm{V̂}\left[\left|L\rangle \langle R\right|+\left|R\rangle \langle L\right|\right]$$

where ${Ĥ}_{1},{Ĥ}_{2}$ and *V̂* depend on the ”nuclear” coordinate and |*R*⟩ and |*L*⟩ represent the two electronic states. To prevent any misunderstanding, henceforth, in this Letter, we refer to *V̂* as the nonadiabatic coupling, which should be distinguished from the nonadiabatic functions appearing in the adiabatic representation, which is not considered in this Letter. The matrix representation of the ”nuclear” Hamiltonian in the diabatic electronic basis set is

2 $$Ĥ=\left(\begin{array}{ll} {Ĥ}_{1} & \mathrm{V̂}\\ \mathrm{V̂} & {Ĥ}_{2} \end{array}\right)$$

We assume that each of the diabatic Hamiltonians has discrete eigenstates with real normalized eigenfunctions:

3 $${Ĥ}_{i}|\psi_{ij}\rangle =E_{ij}|\psi_{ij}\rangle ,i=1,2$$

where the index *i* relates to the diabatic Hamiltonian and the index *j* is for the *j*th state associated with it. We consider the case where the full Hamiltonian has doublets that are close to each other in energy, much closer than the energy spacing of states in the diabatic Hamiltonians. We denote these two doublet eigenstates of the full Hamiltonian as

4 $$Ĥ|\Psi_{k}\rangle =\varepsilon_{k}|\Psi_{k}\rangle ,k=1,2$$

Our goal is to obtain the energy difference,

5 $$\Delta E_{12}\equiv \left(\varepsilon_{1}-\varepsilon_{2}\right)$$

The matrix representation of the full Hamiltonian using two close in energy diabatic states |ψ_1*j*_⟩ and |ψ_2*k*_⟩ takes the form

6 $$\left\langle Ĥ\right\rangle =\left(\begin{array}{ll} \left\langle \psi_{1j}\left|{Ĥ}_{1}\right|\psi_{1j}\right\rangle & \left\langle \psi_{1j}\left|\mathrm{V̂}\right|\psi_{2k}\right\rangle \\ \left\langle \psi_{1j}\left|V\right|\psi_{2k}\right\rangle & \left\langle \psi_{2k}\left|{Ĥ}_{2}\right|\psi_{2k}\right\rangle \end{array}\right)=\left(\begin{array}{ll} E_{1j} & V_{jk}\\ V_{jk} & E_{2k} \end{array}\right)$$

This is a Hermitian Hamiltonian so it has two real eigenvalues,

7 $$\lambda_{\pm }=\frac{\left(E_{1j}+E_{2k}\right)}{2}\pm \sqrt{\frac{{\left(E_{1j}-E_{2k}\right)}^{2}}{4}+V_{jk}^{2}}$$

and associated normalized eigenfunctions, which are linear combinations of the respective diabatic eigenfunctions.

Thus far, there is nothing new. Especially if the two diabatic Hamiltonians are mirror images, the two states are the well-known symmetric and antisymmetric combinations of the eigenstates of the diabatic Hamiltonians. The two-state approximation has been used previously, for example, for the study of tunneling splitting in the excited states of HF dimers.^2,3^ It is at this point though that we introduce some novel elements. One may ask what is the most accurate two-state approximation, or in different words do the diabatic states have to be eigenstates of the respective diabatic Hamiltonians? The answer is yes since one notes that

8 $$\lambda_{+}+\lambda_{-}=E_{1j}+E_{2k}$$

and the best approximation would minimize this sum. The variational principle assures us that the eigenstates of the diabatic Hamiltonians minimize the respective diabatic energies.

A second question is this: what are the conditions necessary for such a two-state approximation to be accurate? If, within the two state-approximation, the uncertainty in the average energy of the doublet (defined as $\sqrt{\langle H^{2}\rangle -\langle H\rangle^{2}}$ , where the mean is given with respect to one of the two states) is greater than the energy splitting between doublets, then the two-state approximation will no longer be valid. This implies, through the Weinstein relation,^21^ that a rough criterion for the validity is that the magnitude of the (constant) nonadiabatic coupling *V* is less than the energy distance between adjacent doublets. If we consider the symmetric case, that is *E*_1*j*_ = *E*_2*j*_ then as seen from eq 7 and noted by Miller,^7^ the energy splitting is just $2\left\langle \psi_{1j}\left|\mathrm{V̂}\right|\psi_{2j}\right\rangle$ , and it is linear in the nonadiabatic coupling. As shown in the Supporting Information, using a four-state diagonalization will change the energy splitting by a term that is at least of the order of ${\left\langle \psi_{1j}\left|\mathrm{V̂}\right|\psi_{2j}\right\rangle }^{2}$ . If the energy splitting is much smaller than the energy difference between adjacent states of the diabatic Hamiltonians, that is, if

$$\left|\left\langle \psi_{1j}\left|\mathrm{V̂}\right|\psi_{2j}\right\rangle \right|\ll E_{1j}-E_{i,j-1},E_{1,j+1}-E_{i,j}$$ 9

then the correction to the two-state energy splitting for the ground-state doublets, which would come from increasing the size of the basis set, is

10 $$\Delta E_{0}=2V_{01}-\frac{V_{01}^{2}}{\left[\left(E_{1}+V_{11}\right)-\left(E_{0}+V_{00}\right)\right]}+O\left(V_{01}^{4}\right)$$

where *V*_01_ = $\left\langle \psi_{10}\left|\mathrm{V̂}\right|\psi_{20}\right\rangle$ , etc. This correction is small and typically negligible. In the semiclassical limit as well as two other examples, we will see that this correction is even exponentially small. When the energy splitting is caused by tunneling, the conditions in eq 9 are typically obeyed, justifying the use of the two-state approximation.

The two-state approximation allows for derivation of analytic formulas for the tunneling splitting, which are not necessarily limited to the ground-state doublet. As a first example, we consider constant nonadiabatic coupling and symmetric diabatic harmonic oscillators, such that the diabatic Hamiltonians are

11 $$H_{1}=\frac{p^{2}}{2M}+\frac{M\omega^{2}}{2}{\left(q+q_{0}\right)}^{2}$$

12 $$H_{2}=\frac{p^{2}}{2M}+\frac{M\omega^{2}}{2}{\left(q-q_{0}\right)}^{2}$$

The normalized eigenfunctions of the harmonic oscillator are well-known. As detailed in the Supporting Information, the energy splitting for the *n*th doublet obtained from the two-state approximation is

13 $$\Delta E_{n}=2V\langle \varphi_{n,-}|\varphi_{n,+}\rangle =2V\;\exp\left(-\theta \right)L_{n}\left(2\theta \right)$$

where the reduced “action” θ is

14 $$\theta =\frac{M\omega q_{0}^{2}}{\hbar }$$

and *L*_*n*_(*x*) is the *n*th Laguerre polynomial. We have checked the validity of eq 13 numerically using a discrete variable representation (DVR) code for a system with a reduced action θ = 12 and found that when the magnitude of the nonadiabatic coupling is given as *V* ≤ *ℏ*ω, the numerical solution agrees with the analytic one reasonably well, as may be seen from the data given in Table 1.

**Table 1:** Ground-State Tunneling Splitting Energies for Diabatic Symmetric Harmonic Potentials

ω	*q*_0_	*V*	θ	Δ*E*_0_ (DVR)	Δ*E*_0_ (analytic)
0.1	6	0.1	3.6	0.0069699	0.0054647
0.15	6	0.1	5.4	0.0010099	0.00090332
0.25	6	0.1	9	2.5441 × 10^–5^	2.4682 × 10^–5^
0.3	6	0.1	10.8	4.1565 × 10^–6^	4.0799 × 10^–6^
0.2	5	0.1	5	0.0014384	0.0013476
0.2	6	0.1	7.2	0.00015766	0.00014932
0.2	7	0.1	9.8	1.1597 × 10^–5^	1.1090 × 10^–5^

Note the exponential dependence of the splitting on the reduced action θ. Since the *n*th Laguerre polynomial will have a contribution of order θ^*n*^, the energy splitting will increase as one goes up the doublet ladder. This increase will not necessarily invalidate the two-state approximation. From the asymptotic relation^22^

15 $$\exp\left(-\theta \right)L_{n}\left(2\theta \right)≃\frac{1}{\sqrt{\pi }}{\left(2n\theta \right)}^{-1/4}\;\cos\left(2\sqrt{2n\theta }-\frac{\pi }{4}\right)+O\left(n^{-3/4}\right)$$

we see that, even for highly excited doublets, the energy splitting will typically remain small, since the magnitude of θ relevant to chemistry is usually larger than 5 and the condition for the validity of the two-state approximation holds. Thus, for a large range of parameters, the analytic result for the energy splitting (eq 13) is valid for any *n*. This is exemplified in Figure 1, where, for low values of *n*, the splitting increases almost exponentially with increasing *n*, as would also be expected from a semiclassical estimate, but it saturates around *n* = 6 where the energy is roughly the crossing point energy. For larger *n*, the overlap between the diabatic states remains of the order of 10^–2^.

**Figure 1:**
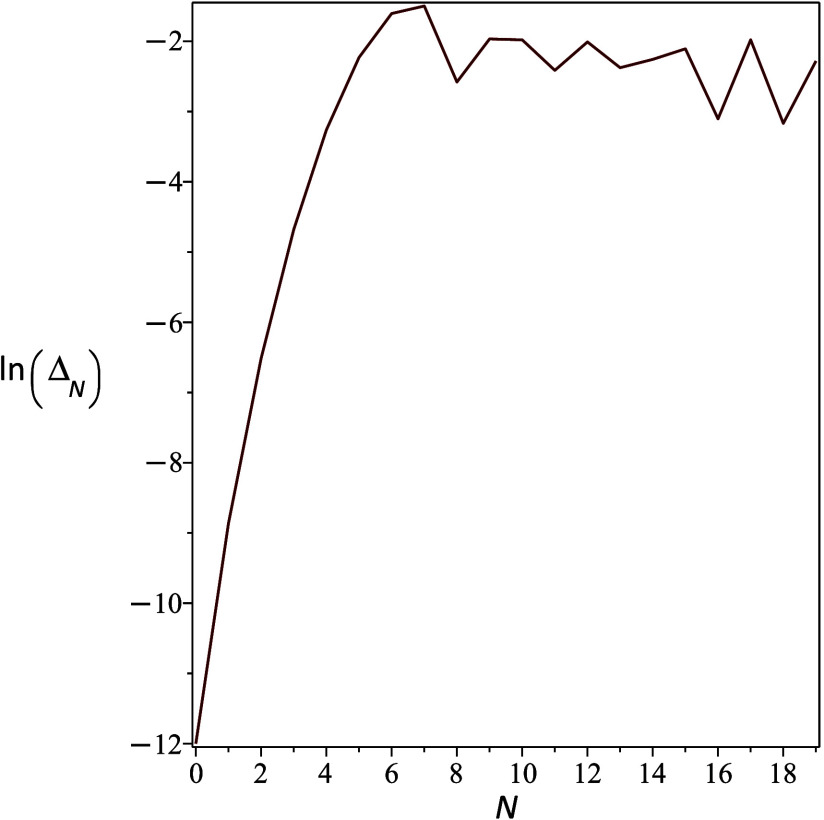
Dependence of the natural logarithm of the symmetric tunneling splitting on the vibrational state. For this model, we used θ = 12 and *V* = 1. *N* denotes the quantum number of the diabatic harmonic state.

The harmonic model is readily extended to the case of coordinate dependent coupling. For example, if the nonadiabatic coupling is Gaussian *V*(*q*) = *V* exp­(−α*q* ^2^), then, as shown in the Supporting Information, the first few doublet splittings are

16 $$\Delta E_{0}=\frac{2V\;\exp\left(-\theta \right)}{\sqrt{\left(1+\mathrm{\alpha ̃}\right)}}$$

17 $$\Delta E_{1}=\Delta E_{0}\left[\frac{1}{\left(1+\mathrm{\alpha ̃}\right)}-2\theta \right]$$

18 $$\Delta E_{2}=\Delta E_{0}\frac{\left[2+{\mathrm{\alpha ̃}}^{2}-4\theta \left(1+\mathrm{\alpha ̃}\right)\left(2+\mathrm{\alpha ̃}\right)+4\theta^{2}{\left(1+\mathrm{\alpha ̃}\right)}^{2}\right]}{{\left(1+\mathrm{\alpha ̃}\right)}^{2}}$$

where α̃ = ℏα/*M*ω is the reduced Gaussian width of the nonadiabatic coupling. Comparing with the results at a constant coupling (α̃=0) , one notices that the splitting is now smaller, as expected, since the nonadiabatic coupling is localized in space.

It is of interest to use the harmonic model to study the transition from symmetric to asymmetric splitting. To simplify, we consider again constant coupling *V* but the diabatic right potential is shifted in energy by Δ*E*. The energy shift does not affect the wave functions, so that the overlap matrix element is (apart from the factor 2*V*), as given in eq 13. Denoting the symmetric energy splitting of the *n*th doublet as Δλ_*n*_(0), one readily sees from eq 7 that the dependence of the doublet splitting energy on the energy shift is

19 $$\Delta \lambda_{n}\left(\Delta E\right)=\sqrt{\Delta E^{2}+\Delta \lambda_{n}{\left(0\right)}^{2}}$$

This result is valid provided that the shift energy Δ*E* ≪ *ℏ*ω. When tunneling dominates, Δλ_*n*_(0) ≪ 1 so that even a rather small shift Δ*E* moves us from a linear dependence of the energy splitting on the nonadiabatic coupling *V* to a quadratic dependence. This is shown in Figure 2, where we plot, for the harmonic model, the reduced splitting for the ground state, $\frac{\Delta \lambda_{0}}{\hbar \omega },$ as a function of $\frac{V}{\hbar \omega }$ for a few values of the energy shift $\frac{\Delta E}{\hbar \omega }$ with a dimensionless action θ = 12.

**Figure 2:**
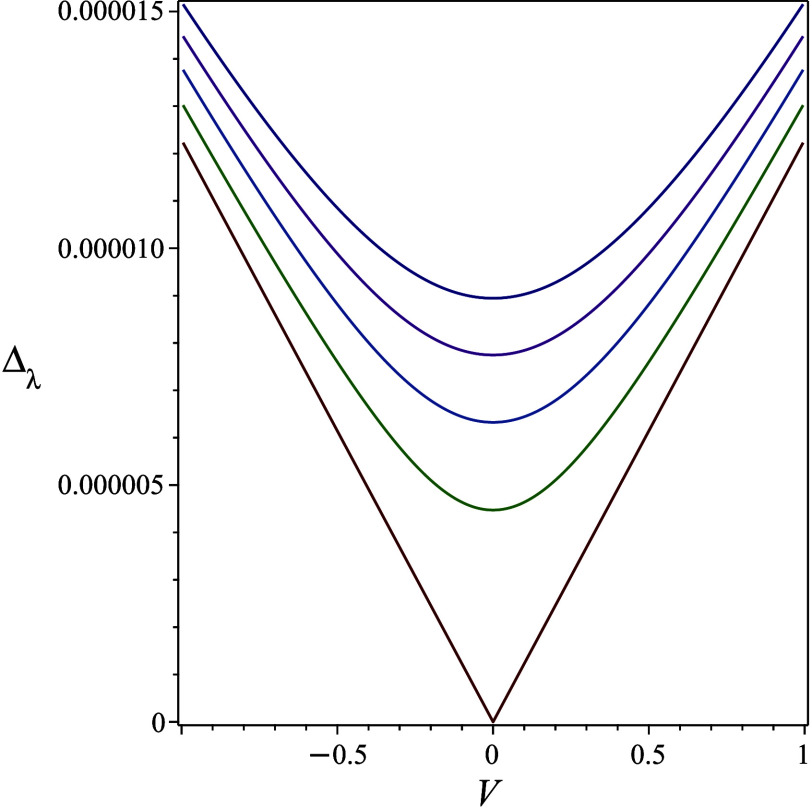
Dependence of the ground-state tunneling splitting on the constant nonadiabatic coupling constant (reduced by *ℏ*ω) for different values of the energy shift. For this model, we used θ = 12, the various lines, from bottom to top, are for $\frac{\Delta E}{\hbar \omega }=0,\sqrt{20}\times 10^{-6},\sqrt{40}\times 10^{-6},\sqrt{60}\times 10^{-6},\sqrt{80}\times 10^{-6}$ . Note the cusp for the lowest line, exemplifying the linear dependence in the nonshifted case.

One may extend the harmonic model to include frequency shifts as well as asymmetry of the diabatic potentials, the effects on the energies are readily obtained with some Gaussian integrals, as shown in the Supporting Information.

Although the harmonic model is instructive, in practice, molecules are not harmonic and it is of interest to see whether it is possible to turn the two-state approximation into a practical method for molecular systems. For this purpose, we consider vibrational perturbation theory (VPT). In contrast to the application of VPT to reaction rates where all that is needed is an expansion of the energy, in terms of the nonlinear contributions,^23–26^ here, the energy splitting is given by the overlap of wave functions, so that VPT must be used for the wave functions. We consider here the symmetric case, so that the left diabatic well is a mirror image of the right diabatic well: *V*_L_(*q*) = *V*_R_(−*q*). The potential of the right well is assumed to have the form

20 VR(q)=Mω22(q−q0)2+V33!(q−q0)3+V44!(q−q0)4≡Mω22(q−q0)2+Vnl(q)

and the first-order wave function on the right side is

21 |ψn⟩R=|φn⟩R+∑k≠n⟨φk|V33!(q−q0)3+V44!(q−q0)4|φn⟩(n−k)ℏω|φk⟩R≡|φn⟩R+|Δφ1,n⟩R

­(where the index 1 in Δφ_1,*n*_ refers to the first-order correction to the wave function) and similarly using the mirror symmetry for the left side wave function. The first-order correction in the nonlinear terms to the tunneling splitting is then

22 ΔEn=2⟨ψn,L|V̂|ψn,R⟩=2⟨φn,L|V̂|φn,R⟩+2⟨Δφ1,n,L|V̂|φn,R⟩+2⟨φn,R|V̂|Δφ1,n,L⟩

One finds (see the Supporting Information) that the first-order right wave function for the *n*th state (the subscript R is implicit) is

$$\psi_{n}\left(q-q_{0}\right)=\varphi_{n}\left(q-q_{0}\right)+\frac{\sqrt{2}}{8}\frac{V_{3}}{\hbar \omega }\left\{{\left(\frac{n\hbar }{M\omega }\right)}^{3/2}\varphi_{n-1}\left(q-q_{0}\right)-{\left[\frac{\left(n+1\right)\hbar }{M\omega_{x}}\right]}^{3/2}\varphi_{n+1}\left(q-q_{0}\right)\right\}+\frac{\sqrt{2}}{73}\frac{V_{3}}{\hbar \omega }{\left(\frac{\hbar }{M\omega }\right)}^{3/2}\left[\sqrt{n\left(n-1\right)\left(n-2\right)}\varphi_{n-3}\left(q-q_{0}\right)-\sqrt{\left(n+3\right)\left(n+2\right)\left(n+1\right)}\varphi_{n+3}\left(q-q_{0}\right)\right]+\frac{1}{96}\frac{V_{4}}{\hbar \omega }{\left(\frac{\hbar }{M\omega }\right)}^{2}\left[\sqrt{n\left(n-1\right)}\left(2n-1\right)\varphi_{n-2}\left(q-q_{0}\right)-\left(3+2n\right)\sqrt{\left(n+1\right)\left(n+2\right)}\varphi_{n+2}\left(q-q_{0}\right)\right]+\frac{1}{384}\frac{V_{4}}{\hbar \omega }{\left(\frac{\hbar }{M\omega }\right)}^{2}\left[\sqrt{\frac{n!}{\left(n-1\right)!}}\varphi_{n-4}\left(q-q_{0}\right)-\sqrt{\frac{\left(n+4\right)!}{n!}}\varphi_{n+4}\left(q-q_{0}\right)\right]$$ 23

For constant nonadiabatic coupling, one then finds that the splitting energy for the *n*th doublet is

$$\Delta E_{n}\;\exp\left(\theta \right)=2VL_{n}\left(2\theta \right)+V\frac{V_{3}}{\hbar \omega }{\left(\frac{\hbar }{M\omega }\right)}^{3/2}\left\{\frac{2}{9}\theta^{3/2}\left[L_{n-3}^{3}\left(2\theta \right)h\left(n-2\right)+L_{n}^{3}\left(2\theta \right)\right]+\sqrt{\theta }\left[\left(n+1\right)L_{n}^{1}\left(2\theta \right)+nL_{n-1}^{1}\left(2\theta \right)h\left(n\right)\right]\right\}+V\frac{1}{24}\frac{V_{4}}{\hbar \omega }{\left(\frac{\hbar }{M\omega }\right)}^{2}\left\{\theta^{2}\left[L_{n-4}^{4}\left(2\theta \right)h\left(n-3\right)-L_{n}^{4}\left(2\theta \right)\right]+2\theta \left[\left(2n-1\right)L_{n-2}^{2}\left(2\theta \right)h\left(n-1\right)-\left(2n+3\right)L_{n}^{2}\left(2\theta \right)\right]\right\}$$ 24

where *h*(*x*) is defined to be the unit step function such that *h*(0) = 0.

Extending this to second order (for the ground-state wave function), we have

25 $${|\psi_{0}\rangle }_{R}={|\varphi_{0}\rangle }_{R}-\underset{k\neq 0}{\sum }\frac{\left\langle \varphi_{k}\left|V_{nl}\left(q-q_{0}\right)\right|\varphi_{0}\right\rangle }{k\hbar \omega }\left(1+\frac{\left\langle \varphi_{0}\left|V_{nl}\left(q-q_{0}\right)\right|\varphi_{0}\right\rangle }{k\hbar \omega }\right){|\varphi_{k}\rangle }_{R}+\underset{k\neq n,l\neq n}{\sum }\frac{\left\langle \varphi_{k}\left|V_{nl}\left(q-q_{0}\right)\right|\varphi_{l}\right\rangle \left\langle \varphi_{l}\left|V_{nl}\left(q-q_{0}\right)\right|\varphi_{0}\right\rangle }{\left(k\cdot l\right)\hbar^{2}\omega^{2}}{|\varphi_{k}\rangle }_{R}-\frac{1}{2}\underset{k\neq 0}{\sum }\frac{{\left\langle \varphi_{k}\left|V_{nl}\left(q-q_{0}\right)\right|\varphi_{0}\right\rangle }^{2}}{k^{2}\hbar^{2}\omega^{2}}{|\varphi_{k}\rangle }_{R}$$

for the right wave function, and for the left, it is the mirror image (ψ_0,L_ = ψ_0,R_(−*q* – *q*_0_)). After some lengthy algebra (see the Supporting Information), noting that the second-order contribution to the tunneling splitting is formally

26 $$\Delta E_{0,2}=2V\langle \Delta \varphi_{1,0,L}|\Delta \varphi_{1,0,R}\rangle +4V\langle \varphi_{0,L}|\Delta \varphi_{2,0,R}\rangle$$

one finds that the ground-state level splitting up to and including second order in the nonlinearity is

$$\frac{\Delta E_{0}}{2V\;\exp\left(-\theta \right)}=1+{\left(\frac{\hbar }{M\omega }\right)}^{3/2}\left(\frac{V_{3}}{\hbar \omega }\right)\left[\left(\frac{1}{9}\right)\theta^{3/2}+\left(\frac{1}{2}\right)\theta^{1/2}\right]-\frac{1}{48}\left(\frac{V_{4}}{\hbar \omega }\right){\left(\frac{\hbar }{M\omega }\right)}^{2}\left(\theta^{2}+6\theta \right)+{\left(\frac{\hbar }{M\omega }\right)}^{3}\left(\frac{V_{3}^{2}}{144\hbar^{2}\omega^{2}}\right)\left[-\frac{29}{6}+\left(\frac{141}{12}\right)\theta +\left(\frac{15}{4}\right)\theta^{2}+\left(\frac{5}{9}\right)\theta^{3}+\left(\frac{1}{6\sqrt{3}}\right)\theta^{3/2}+\left(\frac{9\sqrt{2}}{8}\right)\theta^{1/2}\right]-{\left(\frac{\hbar }{M\omega }\right)}^{7/2}\left(\frac{V_{3}V_{4}}{144\hbar^{2}\omega^{2}}\right)\theta^{1/2}\left[21+\left(\frac{73}{2}\right)\theta +\left(\frac{111}{20}\right)\theta^{2}+\left(\frac{11}{42}\right)\theta^{3}\right]+{\left(\frac{\hbar }{M\omega }\right)}^{4}\left(\frac{V_{4}^{2}}{576\hbar^{2}\omega^{2}}\right)\left[\frac{39}{32}+\left(\frac{63\sqrt{2}-9}{4\sqrt{2}}\right)\theta +\left(\frac{150\sqrt{6}-3}{16\sqrt{6}}\right)\theta^{2}+\left(\frac{7}{6}\right)\theta^{3}+\left(\frac{1}{8}\right)\theta^{4}\right]$$ 27

As an example for the energy splitting of an anharmonic oscillator, we consider a Morse oscillator model such that the right diabatic potential is taken to be

28 $$V_{M,R}\left(q\right)=D{\left\{1-\exp\left[-\alpha \left(q-q_{0}\right)\right]\right\}}^{2}$$

and the left diabatic potential is its mirror image *V*_*M*,L_(*q*) = *V*_*M*,R_(−*q*). Such a model has been considered previously, for example, in ref 27; however, analytic results were not reported. With this construct, the two potentials cross at *q* = 0, where they are exponentially increasing. The eigenvalues and eigenfunctions of the Morse potential are known analytically.^28^ Introducing the dimensionless variables x=αq , $x_{0}=\alpha q_{0}$ , $\lambda =\frac{\sqrt{2MD}}{\alpha \hbar }$ , we then find, using the two-state approximation (see the Supporting Information), that, for constant nonadiabatic coupling, the ground-state energy splitting is given by

29 $$\Delta E_{0}=4V\frac{\left(2\lambda -1\right)}{\Gamma \left(2\lambda \right)}{\left(2\lambda \right)}^{2\lambda -1}\;\exp\left[\left(2\lambda -1\right)x_{0}\right]K_{0}\left[2\lambda \;\exp\left(x_{0}\right)\right]$$

and for the first excited state, it is

$$\Delta E_{1}=\frac{16V\left(2\lambda -3\right)}{\Gamma \left(2\lambda -1\right)}{\left(2\lambda \right)}^{2\lambda -3}\;\exp\left[\left(2\lambda -3\right)x_{0}\right]\left\{\left[{\left(\lambda -1\right)}^{2}+\lambda^{2}\;\exp\left(2x_{0}\right)\right]K_{0}\left[2\lambda \;\exp\left(x_{0}\right)\right]-2\lambda \left(\lambda -1\right)\;\exp\left(x_{0}\right)K_{1}\left[2\lambda \;\exp\left(x_{0}\right)\right]\right\}$$ 30

where *K*_*j*_(*x*) denotes the modified Bessel function of order *j*.

It is then of interest to compare this analytic expression based on the two-state approximation to the numerically estimated ground-state splitting using a DVR method. The second derivative of the Morse potential at the minimum is *V*_2_ = 2*D*α^2^ = *M*ω^2^ and this determines the harmonic frequency. Results are shown in Table 2. In all computations, the mass is taken to be unity and all quantities are given in atomic units. One notices that the numerical and analytic results are in quantitative agreement, as perhaps expected.

**Table 2:** Ground-State Tunneling Splitting Energies for Diabatic Symmetric Morse Potentials

ω	*q*_0_	*D*	*V*	λ	Δ*E*_0_ (DVR)	Δ*E*_0_ (analytic)	θ	Δ*E*_0_ (VPT2)	Δ*E*_0_ (VPT1)
0.1	6	20	0.1	400	0.0054161	0.0042119	3.6	0.004158	0.003996
0.15	6	20	0.1	800/3	0.00061673	0.00055828	5.4	0.0005417	0.0004487
0.25	6	20	0.1	160	8.2798 × 10^–6^	8.0917 × 10^–6^	9	9.4307 × 10^–6^	4.6974 × 10^–6^
0.3	6	20	0.1	400/3	8.9226 × 10^–7^	8.8066 × 10^–7^	10.8	1.8302 × 10^–6^	2.6517 × 10^–6^
0.2	5	20	0.1	200	0.00085367	0.00080565	5	0.0007855	0.0006188
0.2	6	20	0.1	200	0.000072621	0.000069455	7.2	0.00006808	0.00002848
0.2	7	20	0.1	200	3.8209 × 10^–6^	3.6918 × 10^–6^	9.8	4.0702 × 10^–6^	1.8637 × 10^–6^
0.1	6	10	0.1	200	0.0048501	0.0037718	3.6	0.003709	0.003347
0.1	6	30	0.1	600	0.0056815	0.0044209	3.6	0.004378	0.004276

For the Morse potential, the third and fourth derivatives at the right well are *V*_3_ = – 6*D*α^3^, *V*_4_ = 14*D*α^4^, so that the first-order perturbation theory estimate for the ground-state tunneling splitting as obtained from eq 24 is

$$\frac{\Delta E_{0}\left(VPT1\right)}{2V\;\exp\left(-\theta \right)}=1-\frac{1}{\sqrt{\lambda }}\left[\left(\frac{1}{3}\right)\theta^{3/2}+\left(\frac{3}{2}\right)\sqrt{\theta }\right]-\frac{7}{48}\left(\frac{1}{\lambda }\right)\left[\theta^{2}+6\theta \right]$$ 31

and for the first excited state, it is

$$\frac{\Delta E_{1}\left(VPT1\right)}{2V\exp\left(-\theta \right)}=1-2\theta +\frac{1}{3\sqrt{\lambda }}\left(2\theta^{5/2}+14\theta^{3/2}-\frac{45}{2}\sqrt{\theta }\right)+\frac{7}{48}\left(\frac{1}{\lambda }\right)\left(2\theta^{3}+15\theta^{2}-30\theta \right)$$ 32

The second-order estimate for the ground state, as obtained from eq 35, is

$$\frac{\Delta E_{0}\left(VPT2\right)}{2V\;\exp\left(-\theta \right)}=\frac{\Delta E_{0}\left(VPT1\right)}{2V\;\exp\left(-\theta \right)}+\frac{1}{16\lambda }\left[-\frac{29}{6}+\left(\frac{61}{4}\right)\theta +\left(\frac{13}{3}\right)\theta^{2}+\left(\frac{5}{9}\right)\theta^{3}+\left(\frac{1}{6\sqrt{3}}\right)\theta^{3/2}+\left(\frac{9\sqrt{2}}{8}\right)\theta^{1/2}\right]+\frac{7}{48}{\left(\frac{1}{\lambda }\right)}^{3/2}\theta^{1/2}\left[21+\left(\frac{73}{2}\right)\theta +\left(\frac{111}{20}\right)\theta^{2}+\left(\frac{11}{42}\right)\theta^{3}\right]+\frac{49}{576}\left(\frac{1}{\lambda^{2}}\right)\left[\frac{39}{32}+\left(\frac{63\sqrt{2}-9}{4\sqrt{2}}\right)\theta +\left(\frac{150\sqrt{6}-3}{16\sqrt{6}}\right)\theta^{2}+\left(\frac{7}{6}\right)\theta^{3}+\left(\frac{1}{8}\right)\theta^{4}\right]$$ 33

The perturbation theory results for the ground state are also given in Table 2. One notes that, except for very small splitting, the VPT1 theory is quite good, typically with an error of 20% or less, compared to the numerically exact answer. The second-order theory gives a noticeable improvement, with the largest errors found when the reduced action θ is large. It is in these cases that the decay of the wave function in the classically forbidden region is very strong and even the second-order wave function does not always lead to the correct overlap.

In Table 3, we tabulate the first excited-state tunneling splittings, with the same parameters as in Table 2 (the entries for the tunneling splittings are the absolute values). One sees that the analytic result (eq 30) is in very good agreement with the DVR numerical results, justifying the two-state approximation also for an excited state. Moreover, the VPT1 results, are apart from two entries, in reasonable agreement with the numerically exact results, demonstrating that the VPT method is also applicable for an excited state.

**Table 3:** First Excited-State Tunneling Splitting Energies for Symmetric Morse Potentials

ω	*q*_0_	*D*	*V*	λ	Δ*E*_1_ (DVR)	Δ*E*_1_ (analytic)	θ	Δ*E*_1_ (VPT1)
0.1	6	20	0.1	400	0.026378	0.025436	3.6	0.024223
0.15	6	20	0.1	800/3	0.0055284	0.0050517	5.4	0.0037867
0.25	6	20	0.1	160	0.00011753	0.00011480	9	0.00015253
0.3	6	20	0.1	400/3	1.4607 × 10^–5^	1.4413 × 10^–5^	10.8	7.3664 × 10^–5^
0.2	5	20	0.1	200	0.0070108	0.0066665	5	0.0047367
0.2	6	20	0.1	200	0.00085417	0.00081655	7.2	0.00013987
0.2	7	20	0.1	200	5.9873 × 10^–5^	5.7806 × 10^–5^	9.8	6.9257 × 10^–5^
0.1	6	10	0.1	200	0.023946	0.022430	3.6	0.020012
0.1	6	30	0.1	600	0.027468	0.026859	3.6	0.026049

Finally, we consider the semiclassical theory for the tunneling splitting. We assume that the left diabatic potential has at energy *E*_*n*,L_ an outer turning point *q*_*n*,L_ and the right diabatic potential has an inner turning point *q*_*k*,R_ at energy *E*_*k*,R_. The left diabatic potential has a minimum at energy 0 at the point *q*_0,L_ while the right diabatic potential has its minimum at *q*_0,R_, as also sketched in Figure S3 in the Supporting Information. The energy of the minimum of the right diabatic may be different from the left. For the two-state approximation to be valid, we assume that the left and right energies are close to each other, that is, energy *E*_*n*,L_ ≈ *E*_*k*,R_. The semiclassical wave function in the tunneling region of the left diabatic (*q* ≥ *q*_*n*,L_) is^8,29,30^

$$\begin{array}{lll} \psi_{n,L}\left(q\right) & = & \frac{N_{n,L}}{2{\left[2M\left(V_{L}\left(q\right)-E_{n,L}\right)\right]}^{1/4}}\;\exp\left[-\frac{1}{\hbar }\int_{q_{L,n}}^{q}\;dq^{\prime }\sqrt{2M\left(V_{L}\left(q^{\prime }\right)-E_{n,L}\right)}\right]\\ & = & \frac{N_{n,L}}{2\sqrt{\left|p_{n,L}\left(q\right)\right|}}\;\exp\left[-\frac{W_{n,L}\left(q\right)}{\hbar }\right] \end{array}$$ 34

and similarly for the right diabatic potential. The constants *N*_*n*,L_ (and *N*_*k*,R_ for the right diabatic) are normalization constants and are determined by the semiclassical wave function in the classically allowed region. As detailed in the Supporting Information, they are well-approximated as^31^

35 $$\frac{1}{N_{k,R}^{2}}=\frac{T_{k,R}}{4M}$$

and similarly for the left diabatic potential. Here, *T*_*k*,R_ is the classical period of motion on the right diabatic potential at the energy *E*_*k*,R_. The overlap of the two wave functions is then approximated as

36 $$\left\langle \psi_{n,L}\left|\mathrm{V̂}\right|\psi_{k,R}\right\rangle =\int dqV\left(q\right)\;\psi_{n,L}\left(q\right)\psi_{k,R}\left(q\right)=\frac{N_{n,L}N_{k,R}}{4}\int dqV\left(q\right)\;\frac{1}{\sqrt{\left|p_{n,L}\left(q\right)p_{k,R}\left(q\right)\right|}}\times \exp\left[-\frac{W_{n,L}\left(q\right)+W_{k,R}\left(q\right)}{\hbar }\right]$$

and it is maximal when the exponent is minimal in magnitude, indicating that the integration may be estimated using a steepest descent approximation. We note that $\frac{dW_{n,L}\left(q\right)}{dq}=p_{n,L}(q),$ $\frac{dW_{k,R}\left(q\right)}{dq}=-p_{k,R}\left(q\right)$ , so that the maximum overlap occurs when the two momenta are equal in magnitude: *p*_*n*,L_(q*) = *p*_*k*,R_(q*) ≡ *p*(*q*_jk_^*^). The two actions are not necessarily identical at q*; this reflects the two different wave functions which they approximate. Further details of the steepest descent approximation are given in the Supporting Information. Here, we just bring the final result:

37 $$\left\langle \psi_{n,L}\left|\mathrm{V̂}\right|\psi_{k,R}\right\rangle ≃\frac{MV\left(q^{*}\right)}{\sqrt{T_{n}T_{k}}p\left(q^{*}\right)}\sqrt{\frac{\pi \hbar }{\left|\frac{dp_{n,L}\left(q_{nk}^{*}\right)}{dq_{nk}^{*}}-\frac{dp_{k,R}\left(q_{nk}^{*}\right)}{dq_{nk}^{*}}\right|}}\;\times \exp\left[-\frac{W_{n,L}\left(q_{nk}^{*}\right)+W_{k,R}\left(q_{nk}^{*}\right)}{\hbar }\right]$$

In the Supporting Information we also provide details on the application of the semiclassical steepest descent estimate to the case of coupled Morse oscillators. In Tables 4 and 5, we tabulate the tunneling splittings for the first five excited state doublets of two coupled Morse oscillators with ω = 0.25, *q*_0_ = 6.0, *D* = 20, *V* = 0.1 and ω = 0.2, *q*_0_ = 7.0, *D* = 20, and *V* = 0.1, respectively, and with unit mass in both cases. The splittings are computed with eq 37 and compared with the corresponding DVR and VPT results, when available.

**Table 4:** First Six Tunneling Splitting Energies for Symmetric Morse Potentials with ω = 0.25, θ = 9, and *V* = 0.1

*n*	DVR	Δ*E*_*n*_ (semiclassical sd)	Δ*E*_*n*_ (VPT2)	Δ*E*_*n*_ (VPT1)
0	8.2799 × 10^–6^	7.6615 × 10^–6^	9.4307 × 10^–6^	4.6974 × 10^–6^
1	0.00011753	0.00011415	/	0.00015253
2	0.00079529	0.00078551	/	/
3	0.0033848	0.0034053	/	/
4	0.010020	0.010502	/	/
5	0.021420	0.026943	/	/

**Table 5:** First Six Tunneling Splittings Energies for Symmetric Morse Potentials with ω = 0.2, θ = 9.8, and *V* = 0.1

*n*	DVR	Δ*E*_*n*_ (semiclassical sd)	Δ*E*_*n*_ (VPT2)	Δ*E*_*n*_ (VPT1)
0	3.8209 × 10^–6^	3.4897 × 10^–6^	4.0702 × 10^–6^	1.8637 × 10^–6^
1	5.9873 × 10^–5^	5.7349 × 10^–5^	/	6.92574 × 10^–5^
2	0.00044737	0.00043508	/	/
3	0.0021053	0.0020777	/	/
4	0.0069127	0.0070236	/	/
5	0.016458	0.018352	/	/

As one can see, the semiclassical steepest descent estimates are in good agreement with the numerically exact DVR results, and they are more accurate than the VPT1 and VPT2 estimates. This is perhaps not surprising, since the VPT results are based on approximating both the potential and the wave function, while the semiclassical results only come from an approximation to the wave function. For the Morse potential, the semiclassical bound state energies are exact and the approximation is mainly to the wave function.^32,33^

The steepest descent estimate (eq 37) is not identical to the tunneling splitting expression for a symmetric (adiabatic) double well potential (Δ*E*_ad_ = $\frac{2\hbar }{T}\;\exp[-\frac{1}{\hbar }\int_{q_{L}}^{q_{R}}\sqrt{2M\left(V\left(q\right)-E\right)}]$ , where *T* is the period in the well at energy *E* ^7^) but not much different either. The exponential part has in it the same Euclidean action of the instanton orbit as in the adiabatic case, which is the action as one goes from the left to the right turning point. Only when the asymmetry in energy between the two diabatic states is larger than the nonadiabatic matrix element, does one get the asymmetric result with twice the action in the exponent for going from left to right.

The semiclassical result is instructive. On the one hand, the action is composed of two parts, one from each diabatic state, and the two parts match smoothly, as first derived by Cao and Voth^34^ and then used extensively by Richardson and co-workers.^35^ On the other hand, the derivation does not involve a summation over multiple traversals through the classically forbidden region.^7^ The fundamental semiclassical result is half the action in the exponent, inclusion of the full action in the asymmetric case is a result of obtaining the eigenvalues to the 2 × 2 matrix as in eq 7, as suggested by Miller,^7^ but this is not a semiclassical operation. A second aspect is that with the derivation presented here, the semiclassical instanton may be used to obtain estimates of nonadiabatic tunneling splitting in excited states and not only the ground state, as obtained for example, when considering zero temperature limits of thermodynamic partition functions. Future applications will involve multidimensional calculations and one should consider that semiclassical molecular dynamics is well-equipped for such computations, even for ab initio on-the-fly simulations.^36^ More specifically, one of us has recently developed a fully semiclassical approach to eigenfunction calculations^37–41^ that can be readily implemented in the type of framework presented here.

The two-state approach used here for nonadiabatic tunneling suggests that the same approach may also be used in the context of tunneling splitting on a single adiabatic double well potential. Elsewhere, we show that this leads to a result that is identical to the Herring formula. The latter has been implemented successfully for multidimensional systems,^42^ further indicating that the two-state approximation approach is not limited to one-dimensional systems only. At the same time, we note that tunneling splitting on a single adiabatic double well potential without the nonadiabatic coupling is qualitatively different from (the diabatic) nonadiabatic tunneling. In the latter case, when the nonadiabatic coupling constant vanishes there is no tunneling and the splitting is identically 0. If one ignores the nonadiabatic coupling and uses the adiabatic double well potential, then there will be a finite splitting. The differences between ”standard” tunneling and nonadiabatic induced tunneling are, at this point, not fully understood.

The two-state approximation presented in this Letter assumes that the diabatic representation of the system under study is known. This is not trivial. Obtaining the diabatic representation from ab initio Born–Oppenheimer based approaches is not simple and has been discussed in recent years.^43,44^ At the same time, implementing the nonadiabatic coupling within the adiabatic framework is also not straightforward.^43,44^

In summary, this Letter presents an old–new approach to the computation of nonadiabatic tunneling splitting. Vibrational perturbation theory (VPT) has been incorporated and presents the groundwork needed toward a VPT generalization of the theory to many dimensions. Even if the generalization to many dimensions turns out to be difficult in practice, the framework presented here provides a different picture of nonadiabatic tunneling, which we would argue is much simpler than found in other approaches. It all “boils down” to the overlap of the wave functions on the two diabatic states involved.

## Supplementary Material



## Notes

Detailed derivations needed for some of the results presented in the Letter (PDF)
