# Supplementary material for: Theory of Nonadiabatic Tunneling Splitting
Source: J Phys Chem Lett. 2025 May 8;16(20):4844–52. doi: 10.1021/acs.jpclett.5c00443 (PMC12105024; doi:10.1021/acs.jpclett.5c00443)
Supplement: Supplementary file 1 [file jz5c00443_si_001.pdf]

Supporting information for: Theory of nonadiabatic tunneling splitting

Leonardo Raso and Michele Ceotto† and Eli Pollak*,‡

†*Dipartimento di Chimica, Universitá degli Studi di Milano, via C. Golgi 19, 20133 Milano, Italy*

‡*Chemical and Biological Physics Department, Weizmann Institute of Science, 76100 Rehovoth, Israel*

E-mail: eli.pollak@weizmann.ac.il

Preface

In the supporting information we detail the derivations needed to obtain the results presented in the main body of the letter. In Section I, we demonstrate the exponentially small effect of going beyond the two state approximation. In Section II we provide the detailed algebra used to analyze the two state approximation within the framework of diabatic harmonic potentials. In the third section we provide the algebra involved in deriving the perturbation theory expressions for the tunneling splittings. In Section IV we consider the Morse potential tunneling splitting and we end in Section V with the details of the semiclassical analysis.

I. Enlarging the state space gives exponentially small contributions

As mentioned in the paper, the justification for the two state approximation is that under "reasonable" circumstances, the error involved in limiting the basis set to two states is exponentially small. To exemplify this we consider the ground state energy splitting found when enlarging the basis set to include the first excited state for each diabatic well. Using the notation

$$V_{ij} = \langle \psi_{i,L} | \hat{V} | \psi_{j,R} \rangle \tag{1.1}$$

the four state matrix representation of the diabatic Hamiltonian (Eq. 2 in the letter) is a 4x4 matrix

$$\left\langle \hat{H} \right\rangle_{12} = \begin{pmatrix} E_0 & V_{00} & V_{01} & V_{01} \\ V_{00} & E_0 & V_{01} & V_{01} \\ V_{01} & V_{01} & E_1 & V_{11} \\ V_{01} & V_{01} & V_{11} & E_1 \end{pmatrix} \tag{1.2}$$

The eigenvalue equation is readily found to be

$$\begin{aligned} 0 &= [(E_0 - \lambda) - V_{00}] \\ &\cdot \left[[(E_0 - \lambda) + V_{00}] \det \begin{pmatrix} E_1 - \lambda & V_{11} \\ V_{11} & E_1 - \lambda \end{pmatrix} - 4V_{01} \det \begin{pmatrix} V_{01} & V_{01} \\ V_{11} & E_1 - \lambda \end{pmatrix} \right]. \end{aligned} \tag{1.3}$$

This implies that the ground state energy is as in the two state solution

$$\lambda_0 = E_0 - V_{00}. \tag{1.4}$$

The remaining equation may be rewritten as

$$0 = [(E_1 - \lambda) - V_{11}] \left(- [V_{00} + E_0 - \lambda] [(E_1 - \lambda) + V_{11}] + 4V_{01}^2 \right) \tag{1.5}$$

implying that the second excited state energy is the same as if obtained with a two state approximation

$$\lambda_2 = E_1 - V_{11}. \tag{1.6}$$

The remaining quadratic equation is readily solved

$$2\lambda_{\pm} = (E_0 + V_{00} + E_1 + V_{11}) \pm \sqrt{\left[(E_1 + V_{11}) - (E_0 + V_{00})\right]^2 + 4V_{01}^2} \tag{1.7}$$

and this reveals the condition needed for the validity of the two state approximation. Assuming that

$$\left[(E_1 + V_{11}) - (E_0 + V_{00})\right]^2 \gg 4V_{01}^2 \tag{1.8}$$

one finds for the remaining state in the ground state doublet that

$$\lambda_1 = E_0 + V_{00} - \frac{V_{01}^2}{\left[(E_1 + V_{11}) - (E_0 + V_{00})\right]} + O\left(V_{01}^4\right) \tag{1.9}$$

showing that the correction to the two state approximation is of the order of V_{01}^2 as given in Eq. 11 in the main text. This is typically exponentially small not due to the nonadiabatic coupling term but to the very small overlap of the right and left states as also shown in some of the examples considered in the letter. The condition of Eq. 1.8 is not very stringent. It implies that the energy difference between adjacent tunneling doublets is much larger than the matrix element coupling the two states that create the doublet.

II. Symmetric harmonic diabatic states

II.a. Constant nonadiabatic coupling

The two diabatic Hamiltonians are given in Eqs. 12 and 13 in the Letter. The normalized eigenfunctions of the harmonic oscillator are well known[1]

$$\begin{aligned}\langle q|\varphi_{n,\pm}\rangle &\equiv \varphi_{n,\pm}(q) \\ &= \left(\frac{M\omega}{\pi\hbar}\right)^{\frac{1}{4}} \frac{1}{\sqrt{2^n n!}} \exp\left(-\frac{M\omega}{2\hbar}(q\pm q_0)^2\right) H_n\left(\sqrt{\frac{M\omega}{\hbar}}(q\pm q_0)\right)\end{aligned} \tag{2.1}$$

where the H_n's are the Hermite polynomials. When the nonadiabatic coupling is constant, the two state approximation for the energy splitting is

$$\Delta E_n = 2V\langle\varphi_{n,-}|\varphi_{n,+}\rangle. \tag{2.2}$$

To perform the integration, we change variables from q to $x = \sqrt{\frac{M\omega}{\hbar}}q$ so that

$$\begin{aligned}&\langle\varphi_{n,-}|\varphi_{n,+}\rangle \\ =\ & \frac{1}{2^n n!}\frac{1}{\sqrt{\pi}} \exp\left(-\frac{M\omega}{\hbar}q_0^2\right)\int_{-\infty}^{\infty} dx \exp\left(-x^2\right) H_n(x+x_0) H_n(x-x_0) \\ =\ & \exp(-\theta) L_n(2\theta)\end{aligned} \tag{2.3}$$

and this leads to Eq. 14 in the Letter. The reduced action θ is

$$\theta = \frac{M\omega q_0^2}{\hbar}. \tag{2.4}$$

The last line in Eq. 2.3 is Eq. 7.377 in Ref.[2] The Laguerre polynomials are[3]

$$L_n(x) = \sum_{m=0}^{n} (-1)^m \frac{n!}{(n-m)!m!}\frac{x^m}{m!} \tag{2.5}$$

and one readily finds for the first few doublets that

$$\Delta E_0 = |2V| \exp(-\theta) \tag{2.6}$$

$$\Delta E_1 = |2V| \exp(-\theta) [1 - 2\theta] \tag{2.7}$$

$$\Delta E_2 = |2V| \exp(-\theta) \left[1 - 4\theta + 2\theta^2\right] \tag{2.8}$$

$$\Delta E_3 = |2V| \exp(-\theta) \left[1 - 6\theta + 6\theta^2 - \frac{4\theta^3}{3}\right] \tag{2.9}$$

One notes that the splittings are exponentially small, of the order of $\exp(-\theta)$, squaring them will give, as expected, an exponentially small correction term.

II.b. Gaussian nonadiabatic coupling

Here we assume that the coupling has the form

$$V(q) = V \exp\left(-\alpha q^2\right) \tag{2.10}$$

so that the two state approximation for the splitting of the n-th doublet is

$$\Delta E_n = \frac{2V}{\sqrt{\pi} 2^n n!} \exp\left(-\frac{M\omega}{\hbar} q_0^2\right) \int_{-\infty}^{\infty} dx \exp\left(-(1+\tilde{\alpha}) x^2\right) H_n(x + x_0) H_n(x - x_0) \tag{2.11}$$

with

$$\tilde{\alpha} = \frac{\hbar\alpha}{M\omega} \tag{2.12}$$

The integrand is a product of a Gaussian and polynomials so that it is readily evaluated analytically. We did not find an explicit expression valid for all n, but estimated the Gaussian

integrals for the first few states to find (as also given in Eqs. 17-19 in the Letter) that

$$\Delta E_0 = \frac{2V\exp(-\theta)}{\sqrt{(1+\tilde{\alpha})}} \tag{2.13}$$

$$\Delta E_1 = \frac{2V\exp(-\theta)}{\sqrt{1+\tilde{\alpha}}}\left[\frac{1}{(1+\tilde{\alpha})}-2\theta\right] \tag{2.14}$$

$$\Delta E_2 = \frac{V\exp(-\theta)}{\sqrt{(1+\tilde{\alpha})}}\frac{\left[2+\tilde{\alpha}^2-4\theta(1+\tilde{\alpha})(2+\tilde{\alpha})+4\theta^2(1+\tilde{\alpha})^2\right]}{(1+\tilde{\alpha})^2} \tag{2.15}$$

$$\Delta E_3 = \frac{V\exp(-\theta)}{\sqrt{(1+\tilde{\alpha})}} \cdot\frac{\left[-\frac{8}{3}\theta^3(1+\tilde{\alpha})^3+8\theta^2(1+\tilde{\alpha})^2\left(\tilde{\alpha}+\frac{3}{2}\right)-6\theta\left(\tilde{\alpha}^3+3\tilde{\alpha}^2+4\tilde{\alpha}+2\right)+3\tilde{\alpha}^2+2\right]}{(1+\tilde{\alpha})^3}. \tag{2.16}$$

II.c. Asymmetric energy splitting

II.c.1 Energy and spatial minimum shift

In this case the two diabatic potentials take the form

$$V_1(q) = \frac{M\omega^2}{2}(q+q_{01})^2 \tag{2.17}$$

$$V_2(q) = \frac{M\omega^2}{2}(q-q_{02})^2+\Delta E. \tag{2.18}$$

This same model can include the Marcus inverted regime.[4] Let us assume that when the unshifted state has energy $E_n = \left(n+\frac{1}{2}\right)\hbar\omega$, then the two diabatic vibrational states with quantum numbers $n, n-k$ will be in resonance with each other so that the two adjacent states have the energies

$$\lambda_{n\pm}(\Delta E_k) = \left(n+\frac{1}{2}\right)\hbar\omega \pm V\langle\varphi_{n,-}|\varphi_{k,+}\rangle \tag{2.19}$$

Under such circumstances the only change comes from the off diagonal overlap matrix element. Using the reduced coordinate $y = \sqrt{\frac{M\omega}{\hbar}} q$ one finds (Eq. 7.377 in[2])

$$\begin{aligned}
&\langle \varphi_{n,-} | \varphi_{k,+} \rangle \\
= &\frac{1}{\sqrt{\pi 2^n n! 2^k k!}} \int_{-\infty}^{\infty} dy \exp\left(-\frac{1}{2}\left[(y - y_{01})^2 + (y + y_{02})^2\right]\right) H_n(y + y_{01}) H_k(y - y_{02}) \\
= &\frac{\sqrt{2^k k!}}{\sqrt{2^n n!}} \exp\left(-\frac{(y_{01} + y_{02})^2}{4}\right) (3y_{01} - y_{02})^{n-k} L_k^{n-k}\left(\frac{10y_{02}y_{01} - 3(y_{02}^2 + y_{01}^2)}{2}\right) \quad (2.20)
\end{aligned}$$

If the energy shift ΔE is positive and the minima of the diabatic potentials are such $q_{02} \leq -q_{01}$ then we are in the inverted Marcus regime such that $y_{02} = -y_{01} - \Delta y$ and

$$\begin{aligned}
&\langle \varphi_{n,-} | \varphi_{k,+} \rangle \\
= &\frac{\sqrt{2^k k!}}{\sqrt{2^n n!}} \exp\left(-\frac{\Delta y^2}{4}\right) (4y_{01} + \Delta y)^{n-k} L_k^{n-k}\left(-\frac{16y_{01}^2 + 16y_{01}\Delta y + 3\Delta y^2}{2}\right) \quad (2.21)
\end{aligned}$$

II.c.2 Frequency shift

In this case the diabatic potentials are

$$V_1(q) = \frac{M\omega_1^2}{2}(q + q_0)^2 \quad (2.22)$$

$$V_2(q) = \frac{M\omega_2^2}{2}(q - q_0)^2 \quad (2.23)$$

The overlap matrix element for the ground states is

$$\begin{aligned}
&\langle \varphi_{0,-} | \varphi_{0,+} \rangle \\
= &(\omega_1\omega_2)^{\frac{1}{4}} \sqrt{\frac{1}{\pi}} \int_{-\infty}^{\infty} dq \exp\left(-\frac{1}{2}\left[(\omega_1 + \omega_2) q^2 + 2(\omega_2 - \omega_1) q q_0 + (\omega_1 + \omega_2) q_0^2\right]\right) \\
= &\left(1 - \frac{\Delta\omega^2}{4\bar{\omega}^2}\right)^{\frac{1}{4}} \exp\left(-\left(1 - \frac{\Delta\omega^2}{4\bar{\omega}^2}\right)\frac{M\bar{\omega}}{\hbar} q_0^2\right) \quad (2.24)
\end{aligned}$$

with

$$\bar{\omega} = \frac{(\omega_1 + \omega_2)}{2} \tag{2.25}$$

$$\Delta\omega = (\omega_2 - \omega_1) \tag{2.26}$$

Similarly for the first excited state doublet

$$\begin{aligned} & \langle \varphi_{1,-} | \varphi_{1,+} \rangle \\ = & \left(1 - \frac{\Delta\omega^2}{4\bar{\omega}^2}\right)^{\frac{3}{4}} \exp\left(-\left(1 - \frac{\Delta\omega^2}{4\bar{\omega}^2}\right) \frac{M\bar{\omega}}{\hbar}\right) \left(1 - 2\frac{M\bar{\omega}}{\hbar} q_0^2 \left(1 - \frac{\Delta\omega^2}{4\bar{\omega}^2}\right)\right) \end{aligned} \tag{2.27}$$

We then have that the energy splitting is

$$\Delta\lambda_n (\Delta\omega) = \sqrt{\hbar^2 \Delta\omega^2 \left(n + \frac{1}{2}\right)^2 + 4V^2 \langle \varphi_{n,-} | \varphi_{n,+} \rangle^2} \tag{2.28}$$

exemplifying that it does not take much of a frequency shift to induce a quadratic dependence on the nonadiabatic coupling constant.

III. Vibrational perturbation theory

III.a First order in the nonlinearity

We assume that the potential of the right well has the form

$$V_R (q) = \frac{M\omega^2}{2} (q - q_0)^2 + \frac{V_3}{3!} (q - q_0)^3 + \frac{V_4}{4!} (q - q_0)^4 \tag{3.1}$$

In the symmetric case the left well is the mirror image $V_L(q) = V_R(-q)$. First order perturbation theory based on the harmonic oscillator states $|\varphi_n\rangle$ implies that[1]

$$
\begin{aligned}
|\psi_{n,R}\rangle &= |\varphi_n\rangle_R + \sum_{k\neq n} \frac{\langle\varphi_k|\frac{V_3}{3!}(q-q_0)^3 + \frac{V_4}{4!}(q-q_0)^4|\varphi_n\rangle}{(n-k)\hbar\omega}|\varphi_{k,R}\rangle \\
&\equiv |\varphi_{n,R}\rangle + |\Delta\varphi_{1,n,R}\rangle
\end{aligned} \tag{3.2}
$$

and the left state is the mirror image. The first order correction to the tunneling splitting is

$$
\begin{aligned}
\Delta E_n &= 2\langle\psi_{n,L}|\hat{V}|\psi_{n,R}\rangle \\
&= 2\langle\varphi_{n,L}|\hat{V}|\varphi_{n,R}\rangle + 2\langle\Delta\varphi_{1,n,L}|\hat{V}|\varphi_{n,R}\rangle + 2\langle\varphi_{n,R}|\hat{V}|\Delta\varphi_{1,n,L}\rangle
\end{aligned} \tag{3.3}
$$

Noting that

$$
x^3 = \frac{H_3(x) + 6H_1(x)}{8}, x^4 = \frac{H_4(x) + 12H_2(x) + 12}{16} \tag{3.4}
$$

we find for the ground state and the cubic term that

$$
\int_{-\infty}^{\infty} dq\varphi_k(q)\varphi_0(q)q^3 = \frac{1}{4}\sqrt{2}\left(\frac{\hbar}{M\omega}\right)^{\frac{3}{2}}\left(\sqrt{6}\delta_{k3} + 3\delta_{k1}\right) \tag{3.5}
$$

and for the quartic term

$$
\int_{-\infty}^{\infty} dq\varphi_k(q)\varphi_0(q)q^4 = \frac{3}{4}\left(\frac{\hbar}{M\omega}\right)^2\left[\delta_{k4}\frac{4}{\sqrt{6}} + \delta_{k2}\frac{4}{\sqrt{2}} + \delta_{k0}\right] \tag{3.6}
$$

Inserting these results into Eq. 3.2 gives the first order result for the wavefunction (Eq. 24 in the letter)

$$\begin{aligned}
&|\psi_n\rangle \\
= \; & |\varphi_n\rangle + \frac{\hbar V_4}{96\omega^3}\left[\sqrt{n(n-1)}(2n-1)|\varphi_{n-2}\rangle - (3+2n)\sqrt{(n+1)(n+2)}|\varphi_{n+2}\rangle\right] \\
&+\frac{\hbar V_4}{384\omega^3}\left[\sqrt{\frac{n!}{(n-4)!}}|\varphi_{n-4}\rangle - \sqrt{\frac{(n+4)!}{n!}}|\varphi_{n+4}\rangle\right] \\
&+\frac{1}{4!}\frac{V_3}{\omega^2}\sqrt{\frac{2\hbar}{\omega}}\left[\frac{\sqrt{n(n-1)(n-2)}}{3}|\varphi_{n-3}\rangle - \frac{\sqrt{(n+3)(n+2)(n+1)}}{3}|\varphi_{n+3}\rangle\right] \\
&+\frac{1}{8}\frac{V_3}{\omega^2}\sqrt{\frac{2\hbar}{\omega}}\left[n^{\frac{3}{2}}|\varphi_{n-1}\rangle - (n+1)^{\frac{3}{2}}|\varphi_{n+1}\rangle\right] \qquad (3.7)
\end{aligned}$$

One then finds that the first order contribution to the overlap matrix element is

$$\begin{aligned}
&\langle\varphi_{n,L}|\hat{V}|\Delta\varphi_{1,n,R}\rangle \\
= \; & \sum_{k\neq n}\frac{\langle\varphi_k|\frac{V_3}{3!}x^3 + \frac{V_4}{4!}x^4|\varphi_n\rangle}{(n-k)\hbar\omega}\langle\varphi_n\left(x-\sqrt{\theta}\right)|\varphi_k\left(x+\sqrt{\theta}\right)\rangle \\
= \; & \frac{1}{4!}\frac{V_3}{\omega^2}\sqrt{\frac{2\hbar}{\omega}}\left(\frac{1}{3}\sqrt{\frac{n!}{(n-3)!}}I_{n,n-3}(\theta) - \frac{1}{3}\sqrt{\frac{(n+3)!}{n!}}I_{n,n+3}(\theta)\right) \\
&+\frac{1}{4!}\frac{V_3}{\omega^2}\sqrt{\frac{2\hbar}{\omega}}\left(-3(n+1)^{\frac{3}{2}}I_{n,n+1}(\theta) + 3n^{\frac{3}{2}}I_{n,n-1}(\theta)\right) \\
&+\frac{\hbar V_4}{96\omega^3}\left[\sqrt{\frac{n!}{(n-2)!}}(2n-1)I_{n,n-2}(\theta) - \sqrt{\frac{(n+2)!}{n!}}(3+2n)I_{n,n+2}(\theta)\right] \\
&+\frac{\hbar V_4}{384\omega^3}\left[\sqrt{\frac{n!}{(n-3)!}}I_{n,n-4}(\theta) - \sqrt{\frac{(n+4)!}{n!}}I_{n,n+4}(\theta)\right] \qquad (3.8)
\end{aligned}$$

where the overlap integrals $I_{k,n}$ of left and right harmonic oscillator wavefunction integrals are (Eq. 7.377 in[2]).

$$\begin{aligned} I_{k,n} &= \int_{-\infty}^{\infty} dq \varphi_k (q+q_0) \varphi_n (q-q_0) \\ &= \exp(-\theta) \left(\frac{\sqrt{2^n k!}}{\sqrt{2^k n!}} (-1)^{n-k} \theta^{\frac{n-k}{2}} L_k^{n-k}(2\theta) [n \geq k] + \frac{\sqrt{2^k n!}}{\sqrt{2^n k!}} \theta^{\frac{k-n}{2}} L_n^{k-n}(2\theta) [k > n] \right) \end{aligned} \tag{3.9}$$

We then need the explicit expressions for $I_{n,n+4} - I_{n,n-4}$. One readily finds from Eq. 3.9 that

$$I_{n,n}(\theta) = \exp(-\theta) L_n(2\theta) \tag{3.10}$$

$$I_{n,n+1}(\theta) = -\exp(-\theta) \frac{\sqrt{2\theta} L_n^1(2\theta)}{\sqrt{(n+1)}} \tag{3.11}$$

$$I_{n,n+2}(\theta) = \exp(-\theta) \frac{2\theta L_n^2(2\theta)}{\sqrt{(n+1)(n+2)}} \tag{3.12}$$

$$I_{n,n+3}(\theta) = -\exp(-\theta) \frac{2\theta \sqrt{2\theta} L_n^3(2\theta)}{\sqrt{(n+1)(n+2)(n+3)}} \tag{3.13}$$

$$I_{n,n+4}(\theta) = \exp(-\theta) \frac{(2\theta)^2 L_n^4(2\theta)}{\sqrt{(n+1)(n+2)(n+3)(n+4)}} \tag{3.14}$$

and furthermore

$$I_{n,n-4} = I_{m,m+4} \delta_{m,n-4}, \quad I_{n,n-3} = -I_{m,m+3} \delta_{m,n-3}, \quad \text{etc...} \tag{3.15}$$

Putting it all together one finds that the resulting energy splitting is

$$
\begin{aligned}
& \Delta E_n \\
= \; & \Delta E_{n,h} + 4\langle \varphi_{n,L}|\hat{V}|\Delta\varphi_{1,n,R}\rangle \\
= \; & 2V \exp\left(-\theta\right) L_n\left(2\theta\right) \\
& + V\frac{\exp\left(-\theta\right) V_3}{\hbar\omega}\left(\frac{\hbar}{M\omega}\right)^{\frac{3}{2}} \\
& \cdot \left[\frac{2}{9}\theta^{\frac{3}{2}}\left[L^3_{n-3}\left(2\theta\right) h\left(n-2\right) + L^3_n\left(2\theta\right)\right] + \sqrt{\theta}\left[\left(n+1\right) L^1_n\left(2\theta\right) + nL^1_{n-1}\left(2\theta\right) h\left(n\right)\right]\right] \\
& + V\frac{\hbar V_4 \exp\left(-\theta\right)}{24\hbar\omega}\left(\frac{\hbar}{M\omega}\right)^2 \theta \\
& \cdot \left(2\left[\left(2n-1\right) L^2_{n-2}\left(2\theta\right) h\left(n-1\right) - \left(3+2n\right) L^2_n\left(2\theta\right)\right] + \theta\left[L^4_{n-4}\left(2\theta\right) h\left(n-3\right) - L^4_n\left(2\theta\right)\right]\right)
\end{aligned}
\tag{3.16}
$$

and this is the result given in Eq. 25 in the letter.

III.b Second order in the nonlinearity for the ground state doublet

Using the notation

$$V_{nl}\left(q-q_0\right) = \frac{V_3}{3!}\left(q-q_0\right)^3 + \frac{V_4}{4!}\left(q-q_0\right)^4 \tag{3.17}$$

the second order perturbation theory result for the normalized ground state wavefunction based on the harmonic oscillator state $|\varphi_0\rangle$ up to second order is

$$
\begin{aligned}
|\psi_0\rangle_R = \; & |\varphi_0\rangle_R \\
& - \sum_{k\neq 0} \frac{\langle\varphi_k|V_{nl}\left(q-q_0\right)|\varphi_0\rangle}{k\hbar\omega}\left(1 + \frac{\langle\varphi_0|V_{nl}\left(q-q_0\right)|\varphi_0\rangle}{k\hbar\omega}\right)|\varphi_k\rangle_R \\
& + \sum_{k\neq n, l\neq n} \frac{\langle\varphi_k|V_{nl}\left(q-q_0\right)|\varphi_l\rangle\langle\varphi_l|V_{nl}\left(q-q_0\right)|\varphi_0\rangle}{\left(k\cdot l\right)\hbar^2\omega^2}|\varphi_k\rangle_R \\
& - \frac{1}{2}\sum_{k\neq 0} \frac{\langle\varphi_k|V_{nl}\left(q-q_0\right)|\varphi_0\rangle^2}{k^2\hbar^2\omega^2}|\varphi_k\rangle_R
\end{aligned}
\tag{3.18}
$$

for the right wavefunction and for the left it is the mirror image. We then note that

$$\langle\varphi_0|V_{nl}\left(q-q_0\right)|\varphi_0\rangle = \frac{V_4}{32}\frac{\hbar^2}{M^2\omega^2} \tag{3.19}$$

and

$$\begin{aligned}&\langle\varphi_l|V_{nl}\left(q-q_0\right)|\varphi_0\rangle\\ =\;& \frac{V_3}{48}\left(\frac{2\hbar}{M\omega}\right)^{\frac{3}{2}}\left(\sqrt{6}\delta_{l3}+3\delta_{l1}\right)+\frac{V_4}{8}\left(\frac{\hbar}{M\omega}\right)^2\left(\frac{1}{\sqrt{6}}\delta_{l4}+\frac{1}{\sqrt{2}}\delta_{l2}\right)\end{aligned} \tag{3.20}$$

so that

$$\begin{aligned}&\sum_{k\neq 0}\frac{\langle\varphi_k|V_{nl}\left(q-q_0\right)|\varphi_0\rangle}{k\hbar\omega}\left(1+\frac{\langle\varphi_0|V_{nl}\left(q-q_0\right)|\varphi_0\rangle}{k\hbar\omega}\right)|\varphi_k\rangle_R\\ =\;& \frac{V_3\sqrt{3}}{36\hbar\omega}\left(\frac{\hbar}{M\omega}\right)^{\frac{3}{2}}\left(1+\frac{\hbar V_4}{96M^2\omega^3}\right)|\varphi_3\rangle_R+\frac{V_3}{16\hbar\omega}\left(\frac{2\hbar}{M\omega}\right)^{\frac{3}{2}}\left(1+\frac{\hbar V_4}{32M^2\omega^3}\right)|\varphi_1\rangle_R\\ &+\frac{V_4}{16\sqrt{2}\hbar\omega}\left(\frac{\hbar}{M\omega}\right)^2\left[\frac{\sqrt{3}}{6}\left(1+\frac{\hbar V_4}{128M^2\omega^3}\right)|\varphi_4\rangle_R+\left(1+\frac{\hbar V_4}{64M^2\omega^3}\right)|\varphi_2\rangle_R\right]\end{aligned} \tag{3.21}$$

We also note the following integrals

$$\langle\varphi_k|\left(q-q_0\right)^3|\varphi_0\rangle^2 = \left(\delta_{k3}\frac{3}{4}+\delta_{k1}\frac{9}{8}\right) \tag{3.22}$$

$$\langle\varphi_k|\left(q-q_0\right)^4|\varphi_0\rangle^2 = \left(\delta_{k4}\frac{3}{2}+\delta_{k2}\frac{9}{2}\right). \tag{3.23}$$

Using Maple we also find that

$$\left(\frac{M\omega}{\hbar}\right)^{\frac{3}{2}}\langle\varphi_k|\frac{V_3}{3!}(q-q_0)^3|\varphi_3\rangle = \frac{V_3}{3!}\left[\frac{\sqrt{3}}{2}\delta_{k0}+\frac{9\sqrt{6}}{4}\delta_{k2}+6\sqrt{2}\delta_{k4}+\sqrt{15}\delta_{k6}\right] \tag{3.24}$$

$$\left(\frac{M\omega}{\hbar}\right)^{\frac{3}{2}}\langle\varphi_k|\frac{V_3}{3!}(q-q_0)^3|\varphi_1\rangle = \frac{V_3}{3!}\left[\frac{3\sqrt{2}}{4}\delta_{k0}+3\delta_{k2}+\sqrt{3}\delta_{k4}\right] \tag{3.25}$$

$$\left(\frac{M\omega}{\hbar}\right)^{\frac{3}{2}}\langle\varphi_k|\frac{V_3}{3!}(q-q_0)^3|\varphi_4\rangle = \frac{V_3}{3!}\left[\sqrt{3}\delta_{k1}+6\sqrt{2}\delta_{k3}+\frac{15\sqrt{10}}{4}\delta_{k5}\right] \tag{3.26}$$

$$\left(\frac{M\omega}{\hbar}\right)^{\frac{3}{2}}\langle\varphi_k|\frac{V_3}{3!}(q-q_0)^3|\varphi_2\rangle = \frac{V_3}{3!}\left[3\delta_{k1}+\frac{9\sqrt{6}}{4}\delta_{k3}+\frac{\sqrt{30}}{2}\delta_{k5}\right] \tag{3.27}$$

$$\left(\frac{M\omega}{\hbar}\right)^{2}\langle\varphi_k|\frac{V_4}{4!}(q-q_0)^4|\varphi_4\rangle = \frac{V_4}{4!}\left[\frac{\sqrt{6}}{2}\delta_{k0}+7\sqrt{3}\delta_{k2}+\frac{123}{4}\delta_{k4}+\frac{11\sqrt{30}}{2}\delta_{k6}+\sqrt{105}\delta_{k8}\right] \tag{3.28}$$

$$\left(\frac{M\omega}{\hbar}\right)^{2}\langle\varphi_k|\frac{V_4}{4!}(q-q_0)^4|\varphi_2\rangle = \frac{V_4}{4!}\left[\frac{3\sqrt{2}}{2}\delta_{k0}+\frac{39}{4}\delta_{k2}+7\sqrt{3}\delta_{k4}+\frac{3\sqrt{10}}{2}\delta_{k6}\right] \tag{3.29}$$

$$\left(\frac{M\omega}{\hbar}\right)^{2}\langle\varphi_k|\frac{V_4}{4!}(q-q_0)^4|\varphi_1\rangle = \frac{V_4}{4!}\left[\frac{15}{4}\delta_{k1}+\frac{5\sqrt{6}}{2}\delta_{k3}+\frac{\sqrt{30}}{2}\delta_{k5}\right] \tag{3.30}$$

$$\left(\frac{M\omega}{\hbar}\right)^{2}\langle\varphi_k|\frac{V_4}{4!}(q-q_0)^4|\varphi_3\rangle = \frac{V_4}{4!}\left[\frac{5\sqrt{6}}{2}\delta_{k1}+\frac{75}{4}\delta_{k3}+9\sqrt{5}\delta_{k5}+\frac{\sqrt{210}}{2}\delta_{k7}\right] \tag{3.31}$$

The remaining second order contributions to the ground state wavefunction are then:

$$\begin{aligned}
&\sum_{k\neq n, l\neq n}\frac{\langle\varphi_k|V_{nl}(q-q_0)|\varphi_l\rangle\langle\varphi_l|V_{nl}(q-q_0)|\varphi_0\rangle}{(k\cdot l)\hbar^2\omega^2}|\varphi_k\rangle_R \\
= \;& \frac{V_3^2}{144\hbar^2\omega^2}\left(\frac{\hbar}{M\omega}\right)^3\left[\frac{27\sqrt{2}}{4}|\varphi_2\rangle_R+\frac{7\sqrt{6}}{4}|\varphi_4\rangle_R+\frac{\sqrt{5}}{3}|\varphi_6\rangle_R\right] \\
&+\frac{V_3V_4}{144\hbar^2\omega^2}\left(\frac{\hbar}{M\omega}\right)^{\frac{7}{2}}\left[\frac{107\sqrt{2}}{16}|\varphi_1\rangle_R+\frac{211\sqrt{3}}{24}|\varphi_3\rangle_R+\frac{6\sqrt{15}}{5}|\varphi_5\rangle_R+\frac{\sqrt{70}}{28}|\varphi_7\rangle_R\right] \\
&+\frac{V_4^2}{2304}\frac{\hbar^2}{M^4\omega^6}\left[\frac{159\sqrt{2}}{8}|\varphi_2\rangle_R+\frac{291\sqrt{6}}{32}|\varphi_4\rangle_R+\frac{17\sqrt{5}}{4}|\varphi_6\rangle_R+\frac{3\sqrt{70}}{16}|\varphi_8\rangle_R\right]
\end{aligned} \tag{3.32}$$

and

$$
\begin{aligned}
& -\frac{1}{2}\sum_{k\neq 0}\frac{\langle\varphi_k|V_{nl}\left(q-q_0\right)|\varphi_0\rangle^2}{k^2\hbar^2\omega^2}|\varphi_k\rangle_R \\
= \quad & -\frac{\hbar}{2M^3\omega^5}\left[\frac{V_3^2}{144}\left(\frac{1}{3}|\varphi_3\rangle_R+\frac{9}{2}|\varphi_1\rangle_R\right)+\frac{\hbar V_4^2}{1536M\omega}\left(\frac{1}{4}|\varphi_4\rangle_R+3|\varphi_2\rangle_R\right)\right]
\end{aligned}
\tag{3.33}
$$

Putting it all together, we may write down the second order wavefunction as a sum of three contributions, zeroth, first and second order.

$$
|\psi_0\rangle_R=|\varphi_0\rangle_R+|\Delta\varphi_{1,0,R}\rangle+|\Delta\varphi_{2,0,R}\rangle. \tag{3.34}
$$

The zero-th and first order contributions are given in Eq. 3.7. The second order is

$$
\begin{aligned}
& |\Delta\varphi_{2,0,R}\rangle \\
= \quad & \frac{V_3^2}{144\hbar^2\omega^2}\left(\frac{\hbar}{M\omega}\right)^3\left[\frac{27\sqrt{2}}{4}|\varphi_2\rangle_R+\frac{7\sqrt{6}}{4}|\varphi_4\rangle_R+\frac{\sqrt{5}}{3}|\varphi_6\rangle_R-\frac{1}{6}|\varphi_3\rangle_R-\frac{9}{4}|\varphi_1\rangle_R\right] \\
& +\frac{V_3V_4}{144\hbar^2\omega^2}\left(\frac{\hbar}{M\omega}\right)^{\frac{7}{2}}\left[\frac{49\sqrt{2}}{8}|\varphi_1\rangle_R+\frac{35\sqrt{3}}{4}|\varphi_3\rangle_R+\frac{6\sqrt{15}}{5}|\varphi_5\rangle_R+\frac{\sqrt{70}}{28}|\varphi_7\rangle_R\right] \\
& +\frac{\hbar^2V_4^2}{2304M^4\omega^6}\left[\frac{150\sqrt{2}+9}{8}|\varphi_2\rangle_R+\frac{291\sqrt{6}+3}{32}|\varphi_4\rangle_R+\frac{17\sqrt{5}}{4}|\varphi_6\rangle_R+\frac{3\sqrt{70}}{16}|\varphi_8\rangle_R\right]
\end{aligned}
\tag{3.35}
$$

Noting that

$$
\langle\varphi_{0,L}|\varphi_{j,R}\rangle=\exp\left(-\theta\right)\frac{1}{\sqrt{j!}}\left(-1\right)^j\left(2\theta\right)^{\frac{j}{2}} \tag{3.36}
$$

we find that

$$
\begin{aligned}
& \langle \varphi_{0,L} | \Delta\varphi_{2,0,R} \rangle \exp(\theta) \\
= \quad & \frac{V_3^2}{36\hbar^2\omega^2}\left(\frac{\hbar}{M\omega}\right)^3 \left[\frac{81}{24}\theta + \frac{7}{8}\theta^2 + \frac{1}{18}\theta^3 + \frac{1}{12\sqrt{3}}\theta^{\frac{3}{2}} + \frac{9\sqrt{2}}{16}\theta^{\frac{1}{2}}\right] \\
& -\frac{V_3 V_4}{144\hbar^2\omega^2}\left(\frac{\hbar}{M\omega}\right)^{\frac{7}{2}} \left[\frac{49}{4}\theta^{\frac{1}{2}} + \frac{35}{2}\theta^{\frac{3}{2}} + \frac{12}{5}\theta^{\frac{5}{2}} + \frac{1}{21}\theta^{\frac{7}{2}}\right] \\
& +\frac{V_4^2}{4608}\left(\frac{\hbar}{M\omega}\right)^4 \frac{1}{\hbar^2\omega^2}\left[\frac{75\sqrt{2}-9}{\sqrt{2}}\theta + \frac{144\sqrt{6}-3}{4\sqrt{6}}\theta^2 + \frac{17}{3}\theta^3 + \frac{1}{4}\theta^4\right]
\end{aligned}
\tag{3.37}
$$

Noting the fundamental overlap

$$
\begin{aligned}
\langle \varphi_{j,L} | \varphi_{k,R} \rangle &= \frac{1}{\sqrt{\pi}}\frac{(-1)^j}{\sqrt{2^j j! 2^k k!}} \exp(-\theta) \int_{-\infty}^{\infty} dx \exp\left(-x^2\right) H_k(x - x_0) H_j(x + x_0) \\
&= \frac{\sqrt{k!}}{\sqrt{j!}} (-1)^j \exp(-\theta)(2\theta)^{\frac{j-k}{2}} L_k^{j-k}(2\theta), \quad k \le j
\end{aligned}
\tag{3.38}
$$

we find that

$$
\begin{aligned}
& \langle \Delta\varphi_{1,0,L} | \Delta\varphi_{1,0,R} \rangle \exp(\theta) \\
= \quad & \frac{V_3^2}{36\hbar^2\omega^2}\left(\frac{\hbar}{M\omega}\right)^3 \frac{1}{4}\left[5\theta + 2\theta^2 + \frac{4}{9}\theta^3 - \frac{29}{6}\right] \\
& +\frac{V_4 V_3}{288\hbar^2\omega^2}\left(\frac{\hbar}{M\omega}\right)^{\frac{7}{2}} \theta^{\frac{1}{2}}\left[7 - 3\theta - \frac{3}{2}\theta^2 - \frac{1}{3}\theta^3\right] \\
& +\frac{V_4^2}{2304\hbar^2\omega^2}\left(\frac{\hbar}{M\omega}\right)^4 \left[\frac{39}{8} - 12\theta + \frac{3}{2}\theta^2 - \theta^3 + \frac{1}{4}\theta^4\right]
\end{aligned}
\tag{3.39}
$$

Adding all the contributions we get that the level splitting up to and including second order is

$$\begin{aligned}
&\frac{\Delta E_0}{2V\exp\left(-\theta\right)} \\
= \quad &\left[1+\left(\frac{\hbar}{M\omega}\right)^{\frac{3}{2}}\frac{V_3}{\hbar\omega}\left(\frac{1}{9}\theta^{3/2}+\frac{1}{2}\theta^{1/2}\right)-\frac{1}{48}\frac{V_4}{\hbar\omega}\left(\frac{\hbar}{M\omega}\right)^2\left[\theta^2+6\theta\right]\right] \\
&+\frac{V_3^2}{144\hbar^2\omega^2}\left(\frac{\hbar}{M\omega}\right)^3\left[-\frac{29}{6}+\frac{141}{12}\theta+\frac{15}{4}\theta^2+\frac{5}{9}\theta^3+\frac{1}{6\sqrt{3}}\theta^{\frac{3}{2}}+\frac{9\sqrt{2}}{8}\theta^{\frac{1}{2}}\right] \\
&-\frac{V_4V_3}{144\hbar^2\omega^2}\left(\frac{\hbar}{M\omega}\right)^{\frac{7}{2}}\theta^{\frac{1}{2}}\left[21+\frac{73}{2}\theta+\frac{111}{20}\theta^2+\frac{11}{42}\theta^3\right] \\
&+\frac{V_4^2}{576\hbar^2\omega^2}\left(\frac{\hbar}{M\omega}\right)^4\left[\frac{39}{32}+\frac{63\sqrt{2}-9}{4\sqrt{2}}\theta+\frac{150\sqrt{6}-3}{16\sqrt{6}}\theta^2+\frac{7}{6}\theta^3+\frac{1}{8}\theta^4\right]
\end{aligned} \tag{3.40}$$

and this is the final result given in Eq. 28 in the letter.

IV. Symmetric Morse oscillators with constant nonadiabatic coupling

The Morse oscillator potential is either

$$V_{M,+}\left(q\right)=D\left(1-\exp\left[-\alpha\left(q-q_0\right)\right]\right)^2 \tag{4.1}$$

or

$$V_{M,-}\left(q\right)=D\left(1-\exp\left[-\alpha\left(q+q_0\right)\right]\right)^2 \tag{4.2}$$

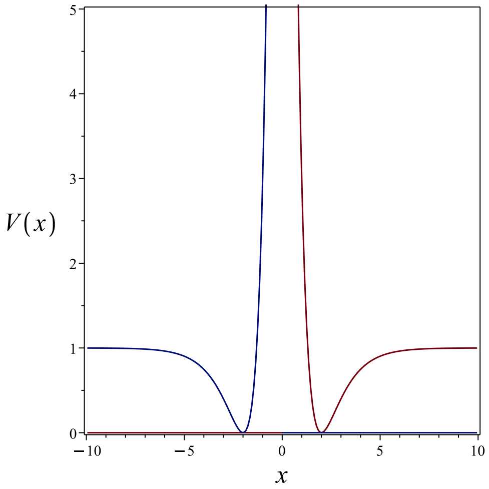

Figure 1: Plot of diabatic Morse potentials - case 1 - demonstrating a tunneling region under the exponentially repulsive parts of the diabatic potentials. The units are dimensionless.

Since the potential is not symmetric about the minimum we must distinguish between two cases. In case 1 the diabatic Hamiltonians are

$$\hat{H}_{11} = \frac{\hat{p}^2}{2M} + V_{M,+}(q) \tag{4.3}$$

$$\hat{H}_{21} = \frac{\hat{p}^2}{2M} + V_{M,+}(-q) \tag{4.4}$$

so that the eigenfunctions of the diabatic potentials overlap under the exponentially increasing portions of the potentials, as shown in Fig. 1. In the second case we have that

$$\hat{H}_{12} = \frac{\hat{p}^2}{2M} + V_{M,-}(q) \tag{4.5}$$

$$\hat{H}_{21} = \frac{\hat{p}^2}{2M} + V_{M,-}(-q) \tag{4.6}$$

and the eigenfunctions overlap under the soft portion of the diabatic potentials as shown in Fig. 2. In the letter, we study case 1.

The eigenvalues and eigenfunctions of the bound states of the Morse potential are known

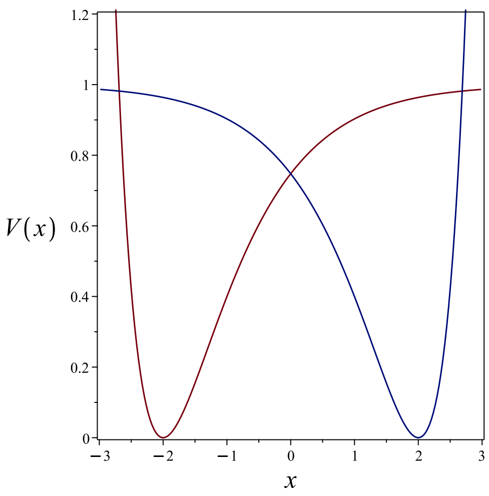

Figure 2: Plot of diabatic Morse potentials - case 2 - demonstrating a tunneling region under the long range attractive parts of the diabatic potentials. The units are dimensionless.

analytically.[5] Introducing the dimensionless variables

$$x = \alpha q, x_0 = \alpha q_0, \lambda = \frac{\sqrt{2MD}}{\alpha \hbar} \tag{4.7}$$

one finds that the eigenfunctions are

$$\varphi_n(z) = N_n z^{\lambda - n - \frac{1}{2}} \exp\left(-\frac{z}{2}\right) L_n^{2\lambda - 2n - 1}(z) \tag{4.8}$$

with

$$z = 2\lambda \exp\left[-(x - x_0)\right] \tag{4.9}$$

and the normalization is

$$N_n^2 = \frac{n!\,(2\lambda - 2n - 1)}{\Gamma(2\lambda - n)} \tag{4.10}$$

Here Γ denotes the gamma function and

$$L_n^{(\gamma)}(z) = \frac{z^{-\gamma} \exp(z)}{n!} \frac{d^n}{dz^n} \left(z^{n+\gamma} \exp(-z)\right) \tag{4.11}$$

is the generalised Laguerre polynomial.[3]

IV.a Ground state energy splitting

The ground state wavefunction of the "right" diabatic state is thus

$$\varphi_{0,R}(x) = \sqrt{\frac{(2\lambda-1)}{\Gamma(2\lambda)}}\left(2\lambda\exp\left[-(x-x_0)\right]\right)^{\lambda-\frac{1}{2}}\exp\left(-\lambda\exp\left[-(x-x_0)\right]\right) \tag{4.12}$$

while for the left diabatic state $\varphi_{0,L}(x) = \varphi_{0,R}(-x)$. The overlaps are readily found using Eq. 3.547.4 of Ref.:[2]

$$\begin{aligned}&\langle\varphi_{0,R}(x)|\varphi_{0,L}(x)\rangle \\ =\ & 2\frac{(2\lambda-1)}{\Gamma(2\lambda)}(2\lambda)^{2\lambda-1}\exp\left[(2\lambda-1)x_0\right]K_0\left[2\lambda\exp(x_0)\right]\end{aligned} \tag{4.13}$$

where $K_0(x)$ is the modified Bessel function. We thus get that the ground state splitting is

$$\Delta E_0 = 4V\frac{(2\lambda-1)}{\Gamma(2\lambda)}(2\lambda)^{2\lambda-1}\exp\left[(2\lambda-1)x_0\right]K_0\left[2\lambda\exp(x_0)\right] \tag{4.14}$$

and this is Eq. 30 in the letter.

IV.b The first excited state

The first excited state wavefunction of the "right" diabatic state is

$$\begin{aligned}\varphi_{1,R}(z) =\ & \sqrt{\frac{(2\lambda-3)}{\Gamma(2\lambda-1)}}(2\lambda)^{\lambda-\frac{3}{2}}\exp\left[-\left(\lambda-\frac{3}{2}\right)(x-x_0)\right] \\ & \exp\left(-\lambda\exp\left[-(x-x_0)\right]\right)\left[2\lambda-2-2\lambda\exp\left[-(x-x_0)\right]\right]\end{aligned} \tag{4.15}$$

and the left state is its mirror image. The overlap is then found to be

$$\begin{aligned}
&\langle \varphi_{1,R}(x) | \varphi_{1,L}(x) \rangle \\
= \ &\frac{8(2\lambda-3)}{\Gamma(2\lambda-1)} (2\lambda)^{2\lambda-3} \exp\left[(2\lambda-3)x_0\right] \\
&\cdot \left(\left[\lambda^2 \exp(2x_0) + (\lambda-1)^2\right] K_0(2\lambda \exp(x_0)) - 2\lambda(\lambda-1)\exp(x_0) K_1(2\lambda\exp(x_0)) \right)
\end{aligned} \tag{4.16}$$

so we find that the first excited doublet splitting is

$$\begin{aligned}
&\Delta E_1 \\
= \ &\frac{16V(2\lambda-3)}{\Gamma(2\lambda-1)} (2\lambda)^{2\lambda-3} \exp\left[(2\lambda-3)x_0\right] \\
&\left(\left[(\lambda-1)^2 + \lambda^2 \exp(2x_0)\right] K_0\left[2\lambda \exp(x_0)\right] - 2\lambda(\lambda-1)\exp(x_0) K_1\left[2\lambda\exp(x_0)\right] \right)
\end{aligned} \tag{4.17}$$

V. Semiclassical two state theory

To carry out the semiclassics one has to be rather careful about connection formulae and normalization. We assume that the left diabatic potential has at energy $E_{L,n}$ an outer turning point $q_{L,n}$ and the right diabatic potential has an inner turning point $q_{R,k}$ at energy $E_{R,k}$. Furthermore we assume that the left diabatic potential has a minimum at energy 0 at the point $q_{0,L}$ while the right diabatic potential has its minimum at $q_{0,R}$. The symmetric case is sketched in Fig. 3. The energy of the minimum of the right diabatic may be different from the left. However for the two state approximation to be valid we assume that the left and right energies are close to each other, that is, energy $E_{n,L} \sim E_{k,R}$. The semiclassical

wavefunction in the tunneling region of the left diabatic ($q \geq q_{L,n}$) is[6,7]

$$\psi_{n,L}(q) = \frac{N_{n,L}}{2\left[2M\left(V_L(q) - E_{n,L}\right)\right]^{\frac{1}{4}}} \exp\left[-\frac{1}{\hbar}\int_{q_{n,L}}^{q} dq' \sqrt{2M\left(V_L(q') - E_{n,L}\right)}\right]$$
$$\equiv \frac{N_{n,L}}{2\sqrt{|p_{n,L}(q)|}} \exp\left[-\frac{W_{n,L}(q)}{\hbar}\right] \tag{5.1}$$

and for the right diabatic potential with $q \leq q_{R,k}$ it is

$$\psi_{k,R}(q) = \frac{N_{k,R}}{2\left[2M\left(V_R(q) - E_{k,R}\right)\right]^{\frac{1}{4}}} \exp\left[-\frac{1}{\hbar}\int_{q}^{q_{k,R}} dq' \sqrt{2M\left(V_R(q') - E_{k,R}\right)}\right]$$
$$\equiv \frac{N_{k,R}}{2\sqrt{|p_{k,R}(q)|}} \exp\left[-\frac{W_{k,R}(q)}{\hbar}\right] \tag{5.2}$$

The constants $N_{n,L}$ and $N_{k,R}$ are normalization constants and are determined by the semi-classical wavefunction in the classically allowed region, as discussed below. The overlap of the two wavefunctions is then approximated as:

$$\langle\psi_{n,L}|\hat{V}|\psi_{k,R}\rangle$$
$$= \int dq V(q)\, \psi_{n,L}(q)\, \psi_{k,R}(q)$$
$$= \frac{N_{n,L}N_{k,R}}{4}\int dq V(q) \frac{1}{\sqrt{|p_{n,L}(q)\, p_{k,R}(q)|}} \exp\left[-\frac{W_{n,L}(q) + W_{k,R}(q)}{\hbar}\right] \tag{5.3}$$

The overlap is maximal when the exponent is minimal in magnitude. We note that derivatives of the actions with respect to the coordinate are the respective momenta:

$$\frac{dW_{n,L}(q)}{dq} = p_{n,L}(q), \frac{dW_{k,R}(q)}{dq} = -p_{k,R}(q) \tag{5.4}$$

so that the maximum overlap occurs when

$$p_{n,L}(q^*) = p_{k,R}(q^*) \equiv p(q^*_{nk}) \tag{5.5}$$

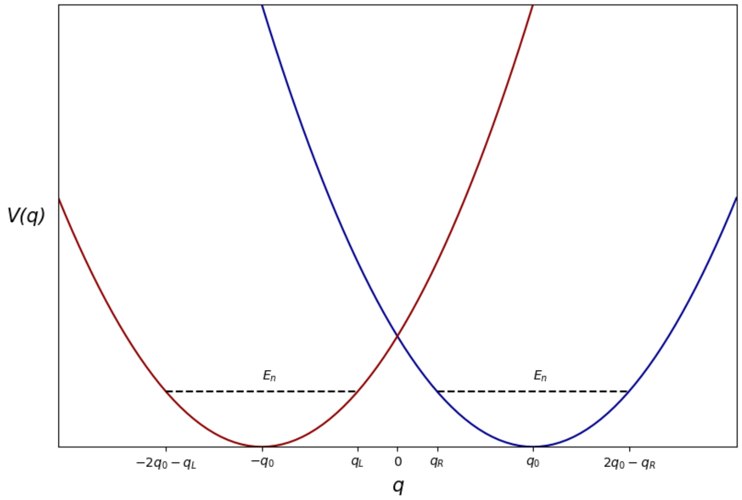

Figure 3: Plot of symmetric diabatic harmonic potentials.

The steepest descent estimate of the overlap integral is then

$$\begin{aligned}&\langle \psi_{n,L}|\hat{V}|\psi_{k,R}\rangle \\ \simeq\quad & \frac{N_{n,L}N_{k,R}}{4}\frac{V\left(q^{*}\right)}{p\left(q^{*}\right)}\sqrt{\frac{\pi\hbar}{\left|\frac{dp_{n,L}\left(q_{nk}^{*}\right)}{dq_{nk}^{*}}-\frac{dp_{k,R}\left(q_{nk}^{*}\right)}{dq_{nk}^{*}}\right|}}\exp\left[-\frac{W_{n,L}\left(q_{nk}^{*}\right)+W_{k,R}\left(q_{nk}^{*}\right)}{\hbar}\right]\end{aligned} \tag{5.6}$$

In the symmetric case, the steepest descent point is found at the crossing point and is independent of the state so that $q_{nk}^{*}=q^{*}$, it is identical to the condition that $V_L\left(q^{*}\right)=V_R\left(q^{*}\right)$ and at the crossing point

$$\frac{dp_{L,n}\left(q^{*}\right)}{dq^{*}} = -\frac{dp_{R,n}\left(q^{*}\right)}{dq^{*}}=\frac{M}{p_n\left(q^{*}\right)}V_L'\left(q^{*}\right) \tag{5.7}$$

so that in the symmetric case we have, up to a normalization constant which is the same for the right and left diabatic potentials and so denoted as N

$$\langle \psi_{n,L}|\hat{V}|\psi_{n,R}\rangle \simeq \frac{N^2}{4}V\left(q^{*}\right)\sqrt{\frac{\pi\hbar p_n\left(q^{*}\right)}{2MV_1'\left(q^{*}\right)}}\exp\left[-\frac{2W_{n,L}\left(q^{*}\right)}{\hbar}\right] \tag{5.8}$$

The missing element is the normalization constant which, as mentioned, is determined only by the classically allowed regions, since in the classically forbidden regions the wavefunction is exponentially small and therefore presumably negligible. So for the right well

$$\frac{1}{N_{k,R}^2} = \int_{q_{k,R}}^{2q_0 - q_{k,R}} dq \frac{1}{\sqrt{2M\left(E_{k,R} - V\left(q\right)\right)}} \sin^2\left[\frac{\pi}{4} + \int_{q_{k,R}}^{q} dq' \frac{1}{\hbar}\sqrt{2M\left(E_{k,R} - V\left(q\right)\right)}\right] \quad (5.9)$$

This is readily simplified, noting that in the classically allowed region $M\dot{q} = p$ and changing variables from coordinate integration to time integration we have

$$\int_{q_{k,R}}^{q} dq' \sqrt{2M\left(E_{k,R} - V\left(q\right)\right)} = 2\int_0^{t_q} dt \left(E_{k,R} - V\left(q_t\right)\right) \equiv 2E_{k,R}\tau_{k,R}\left(t_q\right) \quad (5.10)$$

and this defines the time

$$\tau_{k,R}\left(t_q\right) = \int_0^{t_q} dt \left(1 - \frac{V\left(q_t\right)}{E_{k,R}}\right) \quad (5.11)$$

For a harmonic oscillator the action is linear in the time so that

$$2\int_0^{t_q} dt \left(E_{k,R} - V\left(q\right)\right) = 2E_{k,R}\tau_{k,R}\left(t_q\right). \quad (5.12)$$

Using the identity $\sin^2(x) = [1 - \cos(2x)]/2$ we obtain the desired result

$$\frac{1}{N_{k,R}^2} = \frac{T_k}{4M} + \frac{1}{2M}\int_0^{T_k/2} dt \sin\left[\frac{4E_{k,R}\tau_{k,R}\left(t\right)}{\hbar}\right] \simeq \frac{T_k}{4M} \quad (5.13)$$

where T_k is the classical period of motion at the energy $E_{k,R}$. Typically, the sin term oscillates strongly and therefore it will average out to zero and so may be ignored also when the potential is not harmonic.[8] We thus find that the steepest descent estimate of the overlap

matrix element is

$$\begin{aligned}
&\langle\psi_{n,L}|\hat{V}|\psi_{k,R}\rangle \\
\simeq\; & \frac{MV\left(q^*\right)}{\sqrt{T_nT_k}p\left(q^*\right)}\sqrt{\frac{\pi\hbar}{\left|\frac{dp_{n,L}\left(q^*_{nk}\right)}{dq^*_{nk}}-\frac{dp_{k,R}\left(q^*_{nk}\right)}{dq^*_{nk}}\right|}}\exp\left[-\frac{W_{n,L}\left(q^*_{nk}\right)+W_{k,R}\left(q^*_{nk}\right)}{\hbar}\right]
\end{aligned} \tag{5.14}$$

This is the working expression, as given in Eq. 38 of the Letter.

The key quantities used to compute the semiclassical splitting for the Morse potentials are the period and the action, the first is given by

$$T_k = 2\int_{q_{R,k,-}}^{q_{R,k,+}}\frac{dq}{\sqrt{\frac{2}{M}(E_k - V_R(q))}} = \sqrt{\frac{D}{D-E_k}}\,\frac{2\pi}{M\omega} \tag{5.15}$$

where the integration limits are the classical turning points $V_R(q,k,\pm) = E_k$ and the energy of the k-th state $E_k = \hbar\omega\left[k+\frac{1}{2}-\frac{\hbar\omega}{4D}\left(k+\frac{1}{2}\right)^2\right]$. The integral has been carried out using Eq. 2.266 of Ref..[2] The action in the classically forbidden region from the crossing point q^* to the inner turning point $q_{k,R}$ is

$$\begin{aligned}
W_{k,R}(q^*_k) &= \int_{q*}^{q_{k,R}}\sqrt{2M(V_R(q)-E_k)}\,dq = \frac{2\sqrt{D}}{\omega}\sqrt{V^\ddagger - E_k} \\
&+ \frac{2\sqrt{D}}{\omega}\sqrt{D-E_k}\,\ln\left(\frac{e^{\alpha q_0}\sqrt{4E_kD}}{2\left(E_k - D\left(1-e^{\alpha q_0}\right)-\sqrt{\left(D-E_k\right)\left(V^\ddagger-E_k\right)}\right)}\right) \\
&- \frac{2\sqrt{D}}{\omega}\sqrt{D}\,\ln\left(\frac{\sqrt{D\left(V^\ddagger-E_k\right)}-D(1-e^{\alpha q_0})}{DE_k}\right)
\end{aligned} \tag{5.16}$$

where $V^\ddagger$ is the energy at the crossing point of the diabatic surfaces. The integral has been carried out using Eq. 2.267.1 of Ref..[2] Due to the symmetry of the problem $W_{n,L} = W_{k,R}$.

It is straightforward to test the steepest descent approximation in the case of symmetric harmonic diabatic potentials. For the ground state, in the limit that $\hbar\omega/V^\ddagger \to 0$ one finds

Table 1: Tunneling splitting for a symmetric harmonic oscillator model		
n	ΔE_n (exact)	ΔE_n (sd)
0	$4.1223 \cdot 10^{-10}$	$3.8595 \cdot 10^{-10}$ (6.8%)
1	$1.6077 \cdot 10^{-8}$	$1.5764 \cdot 10^{-8}$ (2.0%)
2	$2.9722 \cdot 10^{-7}$	$2.9490 \cdot 10^{-7}$ (0.8%)
3	$3.4568 \cdot 10^{-6}$	$3.4519 \cdot 10^{-6}$ (0.1%)
4	$2.8296 \cdot 10^{-5}$	$2.8400 \cdot 10^{-5}$ (0.4%)
5	$1.7267 \cdot 10^{-4}$	$1.7425 \cdot 10^{-4}$ (0.9%)
6	$8.1099 \cdot 10^{-4}$	$8.2449 \cdot 10^{-4}$ (1.7%)
7	$2.9801 \cdot 10^{-3}$	$3.0682 \cdot 10^{-3}$ (3.0%)
8	$8.6032 \cdot 10^{-3}$	$9.1135 \cdot 10^{-3}$ (5.9%)
9	0.01934	0.02295 (18.7%)

that the steepest descent prediction for the splitting is $\sqrt{\frac{e}{\pi}} 2V exp(-\theta)$. Since $\sqrt{e/\pi} \sim 0.9302$ this implies an error of 7% only. In the Table, we consider a model in which $\frac{V^\ddagger}{\hbar\omega} = 10$. The column labeled ΔE_n(exact) is based on the analytic two state harmonic result (Eq. 2.3) which we know from separate DVR computations is quite accurate. The column ΔE_n(sd) shows the steepest descent estimates as obtained from Eq. 5.14. The numbers in parentheses show the relative error in percentages. The agreement is very good also for the excited state doublets, justifying the instanton based computation. The error is relatively large for the highest excited state, where by nature the steepest descent approximation starts deteriorating due to the proximity of the two turning points. Conversely, the error is larger for the ground state as compared to the adjacent excited state doublets, due to the nonclassical structure of the wavefunction in the ground state, where the maxima are not at the turning points and the error is close to the theoretical error of 7%.

References

(1) Cohen-Tannoudji, C.; Franck Laloë, B., Quantum Mechanics, Vol. 1, Chap. 5 (J. Wiley and Sons, New York) **1977**.

(2) Gradshteyn, I.S.; Ryzhik, I.M., Table of integrals series and products, (Academic Press, New York) **1980**

(3) Abramowitz, M.; Stegun, I.A., Handbook of mathematical functionswith formulas, graphs, and mathematical tables, (Dover publ.) **1965**, chap. 22.

(4) Marcus, R.; Sutin, N., Electron transfers in chemistry and biology, *Biochim. Biophys. Acta* **1985**, *811*, 265–322.

(5) Dahl, J.P.; Springborg, M., The Morse oscillator in position space, momentum space, and phase space, *J. Chem. Phys.*, **1988**, *88*, 4535-4547.

(6) Child, M.S., Molecular Collision Theory, *Academic Press, London, NY* **1974**, Appendix C.2.

(7) Song, D-Y., Tunneling and energy splitting in an asymmetric double-well potential, *Ann. Phys.*, **2008**, *323* 2991-2999.

(8) Moxhay P.; Rosner, J.L., Semiclassical results on normalization of bound state wavefunctions, *J. Math. Phys.*, **1980**, *21*, 1688.
